# Intrinsic topological Weyl phase transition induced by a magnetostructural transformation in a kagome magnet

**DOI:** 10.1038/s41467-026-71683-7

**Published:** 2026-04-11

**Authors:** Tsung-Han Yang, Satoshi Okamoto, D. Alan Tennant, Michael A. McGuire, Qiang Zhang

**Affiliations:** 1 Neutron Scattering Division, Oak Ridge National Laboratory, Oak Ridge, TN USA; 2 Materials Science & Technology Division, Oak Ridge National Laboratory, Oak Ridge, TN USA; 3 Department of Physics & Astronomy, University of Tennessee, Knoxville, TN USA; 4 Shull Wollan Center, Oak Ridge National Laboratory, Oak Ridge, TN USA; 5 Department of Materials Science and Engineering, University of Tennessee, Knoxville, TN USA

**Keywords:** Topological matter, Magnetic properties and materials, Phase transitions and critical phenomena

## Abstract

Topological phase transitions provide a unique window into the interplay between structure, magnetism, and Weyl physics in magnetic Weyl semimetals. However, realizing an intrinsic Weyl phase transition between two distinct Weyl states near room temperature remains challenging. Here, we demonstrate that a magnetostructural transition effectively induces such a transition in the kagome magnet Mn_3_Ga. High-resolution neutron diffraction, magnetization characterizations and first-principles calculations reveal that Mn_3_Ga undergoes a chiral antiferromagnetic transition below 485 K, followed by a magnetostructural transition to a monoclinic structure with highly canted antiferromagnetic order near room temperature. These cooperative changes in lattice and magnetic symmetries reorganize Weyl nodes, driving a transition from a primary type-II Weyl state to a distinct Weyl state, accompanied by dramatic variations in the anomalous Hall effect and appearance of topological Hall effect. Our findings open a new pathway for discovering novel topological Weyl states and advancing potential spintronic applications.

## Abstract (web summary)

Topological phase transitions between two distinct Weyl topological states have thus far proven difficult to find. Here, using a combination of neutron diffraction, transport measurement and first principles calculations, Yang et al show that a magneto-structural transition in the kagome magnet Mn_3_Ga exhibits such a topological phase transition.

## Introduction

The discovery of topological quantum materials has led to transformative insights into condensed matter physics, with Weyl semimetals emerging as a particularly intriguing class due to their gapless excitations associated with Weyl nodes, band crossings which act as monopoles of Berry curvature in momentum space^1–6^. While early realizations focused on nonmagnetic systems with broken inversion symmetry, magnetic Weyl semimetals have recently garnered significant interest, as the time-reversal symmetry breaking by magnetic order offers additional control over the topological band structure^7–10^. Exploring topological phase transitions provides an excellent opportunity to investigate the intricate coupling among structure, magnetism, and Weyl physics. To date, only a limited number of compounds have been identified to undergo topological phase transitions from one non-Weyl topological state to a Weyl state, primarily driven by external stimuli such as magnetic fields or pressure^11–13^. For example, an applied magnetic field induces the transformation from an antiferromagnetic (AFM) topological insulator to an ideal type-II Weyl state in Mn(Bi_1−*x*_Sb_*x*_)_2_Te_4_^11^. A pressure-induced transition from a magnetic topological insulator to a trivial insulator, and then to a Weyl semimetal, was reported in EuCd_2_As_2_^13^. From the perspective of both fundamental and applied research, it is particularly intriguing yet challenging to explore strategies for inducing intrinsic topological phase transitions between two distinct Weyl states without external stimuli, especially under conditions near room temperature.

**Fig. 1:**
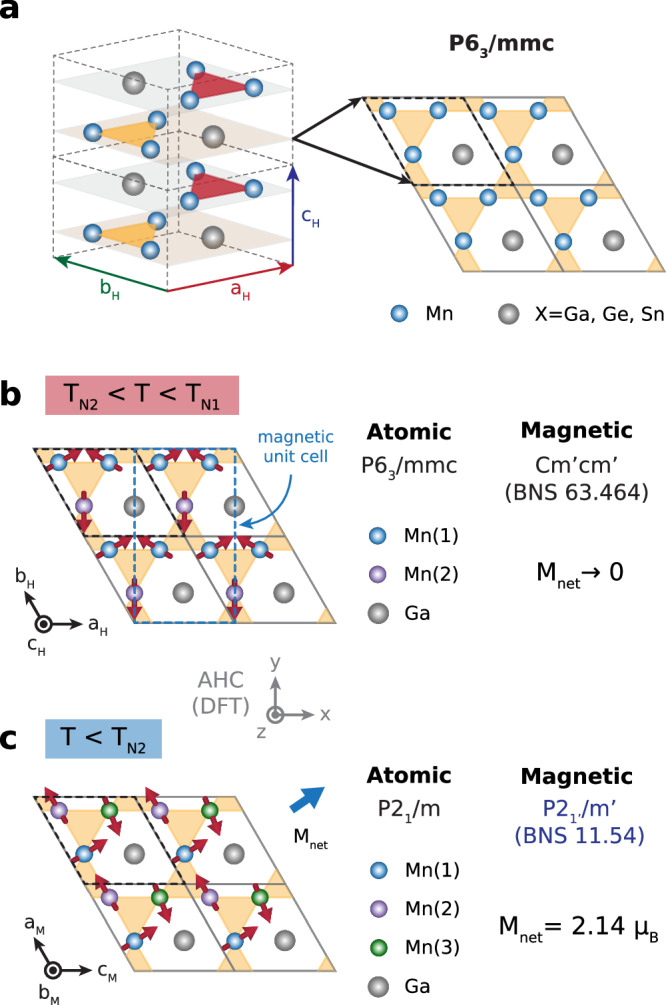
Crystal and magnetic structures of Mn_3_Ga. **a** The high-temperature paramagnetic phase with hexagonal *P*6_3_/*mmc* (No. 194) symmetry, isostructural to Mn_3_Sn and Mn_3_Ge. Mn atoms form two-dimensional kagome layers stacked along the hexagonal *c*_H_ axis. **b** Chiral AFM magnetic structure in the intermediate-temperature phase (*T*_N2_ < *T* < *T*_N1_). While the underlying crystal structure remains a hexagonal structure, magnetic ordering breaks the six-fold rotational symmetry, resulting in an orthorhombic magnetic unit cell (indicated by the blue dashed line), with magnetic space group $C{m}^{{\prime} }c{m}^{{\prime} }$ (BNS 63.464). **c** Low-temperature phase (*T* < *T*_N2_). The crystal symmetry lowers to monoclinic *P*2_1_/*m* (No. 11), accompanied by a reorientation of Mn magnetic moments. The resulting magnetic space group is $P{2}_{{1}^{{\prime} }}/{m}^{{\prime} }$ (BNS 11.54), with a large net ferromagnetic moment of **M**_net_ ≈ 0.71 *μ*_*B*_ /Mn oriented approximately perpendicular to the *a*_M_ axis. The gray axes (*x*, *y*, *z*) define the laboratory frame used to calculate AHC in both phases.

The kagome magnets Mn_3_Sn and Mn_3_Ge, which share the same hexagonal structure as Mn_3_Ga, shown in Fig. 1a, have attracted significant attention^7,14–20^ due to their recent identification as magnetic Weyl semimetals exhibiting chiral magnetic order and remarkable quantum transport properties. In Mn_3_Sn, the interplay between the noncollinear antiferromagnetic order and strong spin-orbit coupling gives rise to significant anomalous and topological Hall effects (AHE and THE)^7,14^. Furthermore, a magnetic inverse spin Hall effect^19^ and a large anomalous Nernst effect^16^ have been discovered in Mn_3_Sn. While in Mn_3_Ge, a similar chiral spin structure combined with Weyl points lying closer to the Fermi level produces an even larger AHE despite its moderate spin-orbit coupling^15^. Compared to the more extensively studied Mn_3_Sn and Mn_3_Ge, which show topological behavior within a high-symmetry hexagonal structure, Mn_3_Ga is distinguished by a temperature-dependent magnetostructural transition (MST)^21–24^, i.e., a coupled crystal and magnetic transition, as shown in Fig. 1b, c. Mn_3_Ga is isostructural with Mn_3_Sn and Mn_3_Ge at high temperatures and has been reported to exhibit a 120^∘^ chiral antiferromagnetic order below *T*_N1_ ≈ 470 K^21^. Upon cooling to the lower MST temperature *T*_N2_ ≈ 120 K^24^ or 170 K^22,23^, Mn_3_Ga undergoes a structural transition, reported as controversial orthorhombic^22,24^ or monoclinic^23^, accompanied by a magnetic reconfiguration that gives rise to a substantial net ferromagnetic moment^22–25^. Previous studies have revealed AHE and THE signatures^24,25^ on polycrystalline Mn_3_Ga, associated with the distinct crystalline and magnetic states in the temperature range *T*_N2_ < *T* < *T*_N1_ and *T* < *T*_N2_, respectively, suggesting a strong correlation between structural changes and topological properties. However, determining the true ground-state structure and spin configuration remains challenging due to the subtlety of simultaneous crystalline and magnetic symmetry lowering. These difficulties have hindered the identification of precise topological states below and above MST in Mn_3_Ga.

In this work, we report that Mn_3_Ga exhibits an intrinsic topological Weyl phase transition between two distinct Weyl states, driven by a magnetostructural transition. Using a combination of high-resolution neutron diffraction, magnetization measurements, and first-principles density functional theory (DFT) calculations, we have determined and validated the distinct crystal and magnetic structures in *T*_N2_ < *T* < *T*_N1_ and *T* < *T*_N2_ in Mn_3_Ga. The MST temperature in our Mn_*x*_Ga (*x* = 2.94(1)) (hereafter referred to as Mn_3_Ga for simplicity), was enhanced to near room temperature (*T*_N2_ ≈ 295 K). Furthermore, we find that two distinct crystalline and magnetic ordered phases can each host Weyl nodes. More importantly, the MST fundamentally alters both the multiplicity and distribution of Weyl nodes, thereby driving a topological Weyl phase transition to a distinct Weyl state accompanied by a modification of the anomalous Hall conductivity (AHC) with the emergence of a non-zero *σ*_*yz*_ component near room temperature. Transport measurements reveal a sign reversal of the anomalous Hall conductivity alongside a significant enhancement in its magnitude below the MST, providing experimental proof of the redistribution of the Berry curvature and a corresponding reconstruction of the Weyl-node manifold. The emerged THE, coexisting with AHE below a lower temperature  ≈ 150 K, highlights a wonderful unification of the real-space Berry phase arising from spin chirality and *k*-space Berry curvature inherent to the band topology. Our results thus demonstrate that the MST provides a compelling pathway for inducing a topological Weyl phase transition in correlated magnetic materials.

## Results

### Chiral antiferromagnetic ordering at *T*_N1_

**Fig. 2:**
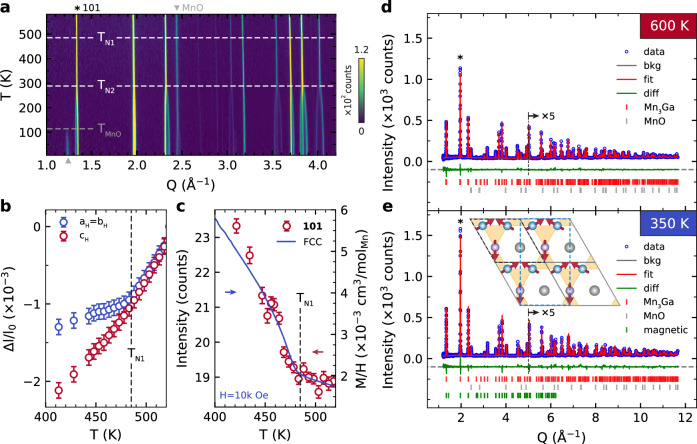
Neutron diffraction and magnetization measurements of the intermediate-temperature phase (*T*_N2_ < *T* < *T*_N1_) in Mn_3_Ga. **a** Neutron diffraction patterns measured from 6 K to 580 K. Black and gray asterisk symbols indicate hexagonal 101 peak and MnO impurity phase. The gray arrow indicates the $\frac{1}{2}\frac{1}{2}\frac{1}{2}$ magnetic Bragg peak of MnO, and the temperature-dependence of $\frac{1}{2}\frac{1}{2}\frac{1}{2}$ intensity is shown in Supplementary Fig. 4. **b** Temperature dependence of normalized lattice parameters. A clear lattice anomaly within the kagome plane (*a*_H_ and *b*_H_) is observed right below *T*_N1_, indicating the onset of spin-lattice coupling. **c** Temperature dependence of field-cooled-cooling (FCC) magnetization and 101 Bragg peak intensity. **d** Rietveld refinement at 600 K confirms that the sample composition is near-stoichiometric Mn_*x*_Ga (*x* = 2.94(1)). The neutron diffraction data are well described by the hexagonal *P*6_3_/*mmc* (No. 194) structure (*Rw* = 0.0727). **e** Rietveld refinement at 350 K indicates no detectable structural phase transition. The magnetic structure is well described by the $C{m}^{{\prime} }c{m}^{{\prime} }$ (BNS 63.464) magnetic space group (*Rw* = 0.0791).

We begin by investigating the magnetic structure of Mn_3_Ga using temperature-dependent neutron diffraction and magnetic characterization measurements (Fig. 2). As shown in Fig. 2a, the colormap presents the overview of phase transitions. At *T*_N1_ = 485 K, the crystal structure retains hexagonal symmetry, but shows a reduced structural symmetry below *T*_N2_ = 295 K, as indicated by the splitting of Bragg peaks. Lattice parameters were extracted from a series of Rietveld refinements against the diffraction data, and Fig. 2b shows that the lattice parameter (*a*_H_ = *b*_H_) exhibits a pronounced anomaly at *T*_N1_, despite the absence of any symmetry-breaking structural transition. The observed *T*_N1_ is slightly higher than those^21,22^ (<470 K) in previous reports. Magnetic susceptibility measurement on a polycrystalline sample (Fig. 2c) shows a distinct anomaly at 485 K, indicating a magnetic transition. Consistently, the intensity of the hexagonal 101 Bragg peak increases gradually upon cooling below *T*_N1_, indicating the feature of a second-order magnetic phase transition, as also shown in Fig. 2c. These results collectively indicate strong spin-lattice coupling at *T*_N1_, even in the absence of a crystallographic symmetry change.

Rietveld refinements against powder neutron diffraction patterns measured above and below *T*_N1_ are presented in Fig. 2d, e. For temperatures above *T*_N1_, the refinements indicate the presence of approximately 5.5 wt% MnO impurity, which was held constant in all subsequent refinements. The nominal Mn_3_Ga phase adopts a hexagonal crystal structure with space group *P*6_3_/*mmc* (No. 194), and the refined chemical composition yields Mn_*x*_Ga (*x* = 2.94(1)), indicating a 2.3(3)% of manganese deficiency relative to ideal stoichiometry. Upon cooling into the intermediate temperature range, *T*_N2_ < *T* < *T*_N1_, the symmetry analysis and refinements confirm the $C{m}^{{\prime} }c{m}^{{\prime} }$ (BNS 63.464) magnetic structure with magnetic propagation vector ***k*** = 0. The refined magnetic structure shows noncollinear spins confined to the kagome planes, resulting in a nearly zero net magnetic moment per unit cell (not symmetry-constrained). In each triangular unit, the three Mn moments are oriented ⊥**a**_H_, ⊥**b**_H_, and ∥(**b**_H_ − **a**_H_), forming a 120^∘^ chiral antiferromagnetic order as shown in the insert of Figs. 2e and 1b. The ordered moment per Mn site is refined to be 2.06(25) *μ*_B_ at 350 K. The refined atomic positions and magnetic moments are listed in Table 1.

**Table 1:** Refined atomic positions and magnetic moments at 350 K (represented in a hexagonal cell)

Atom	*x*	*y*	*z*	Symm. constr.	*M*_*x*_	*M*_*y*_	*M*_*z*_	∣M∣
Mn(1)	0.6722 (3)	0.8361 (5)	0.2500	*m*_*x*_, −*m*_*x*_, 0	−1.18 (17)	1.18 (17)	0.00	2.06 (25)
Mn(2)	0.8361 (7)	0.6722 (3)	0.7500	*m*_*x*_, *m*_*y*_, 0	−1.18 (17)	−2.36 (17)	0.00	2.06 (25)
Ga	0.3333	0.6667	0.7500	–	–	–	–	–

The refinement was performed in the magnetic space group $C{m}^{{\prime} }c{m}^{{\prime} }$ (BNS 63.464), with lattice parameters *a*_H_ = *b*_H_ = 5.4078(9) Å, *c*_H_ = 4.3568(2) Å.

This magnetic structure differs from the 120^∘^ antiferromagnetic order previously reported in Mn_3_Ga, where the moment direction is parallel to the crystalline axes^23^. Refinements using $C{m}^{{\prime} }c{m}^{{\prime} }$ (BNS 63.464) magnetic model provide improved agreement with our neutron diffraction data compared to the previously reported $P{6}_{{3}^{{\prime} }}/{m}^{{\prime} }m{c}^{{\prime} }$ (BNS 194.269) magnetic model based on the early neutron powder diffraction study^23^. The comparison is shown in Supplementary Fig. 2. It is worthwhile pointing out that to determine (***k*** = 0) magnetic order, the high-resolution neutron diffraction data with wide *Q* coverage is essential to obtain the reliable structural parameters, such as the scale factor, atomic positions, thermal parameters, occupancy, etc., for separating the nuclear and magnetic contributions to the same peak and distinguishing different magnetic models^26^. Our DFT calculations reveal that the energy of $C{m}^{{\prime} }c{m}^{{\prime} }$ configuration is −30.002 eV f.u.^−1^, which is lower than −29.996 eV f.u.^−1^ calculated for the previously proposed $P{6}_{{3}^{{\prime} }}/{m}^{{\prime} }m{c}^{{\prime} }$ configuration. This energy difference further validates $C{m}^{{\prime} }c{m}^{{\prime} }$ magnetic structure. Furthermore, we observe weak spontaneous magnetization and a small hysteresis loop for *T*_N2_ < *T* < *T*_N1_, as discussed below. This indicates the presence of a small uncompensated component to the moment in Mn_3_Ga, analogous to the small in-plane net moment along the *a*_H_ axis in Mn_3_Sn^7^ and Mn_3_Ge^15^. Note that this minuscule moment is undetectable by neutron diffraction yet is allowed by the magnetic symmetry $C{m}^{{\prime} }c{m}^{{\prime} }$ (see Table 1). Consequently, the magnetic order in the *T*_N2_ < *T* < *T*_N1_ range is described as a nearly 120^∘^ triangular AFM structure ($C{m}^{{\prime} }c{m}^{{\prime} }$) with a slight canting that produces a weak net moment along the *a*_H_ axis. This configuration is identical to that of iso-structural Mn_3_Ge^27,28^ and aligns with previous DFT reports^29^.

### Magnetostructural phase transition at *T*_N2_

**Fig. 3:**
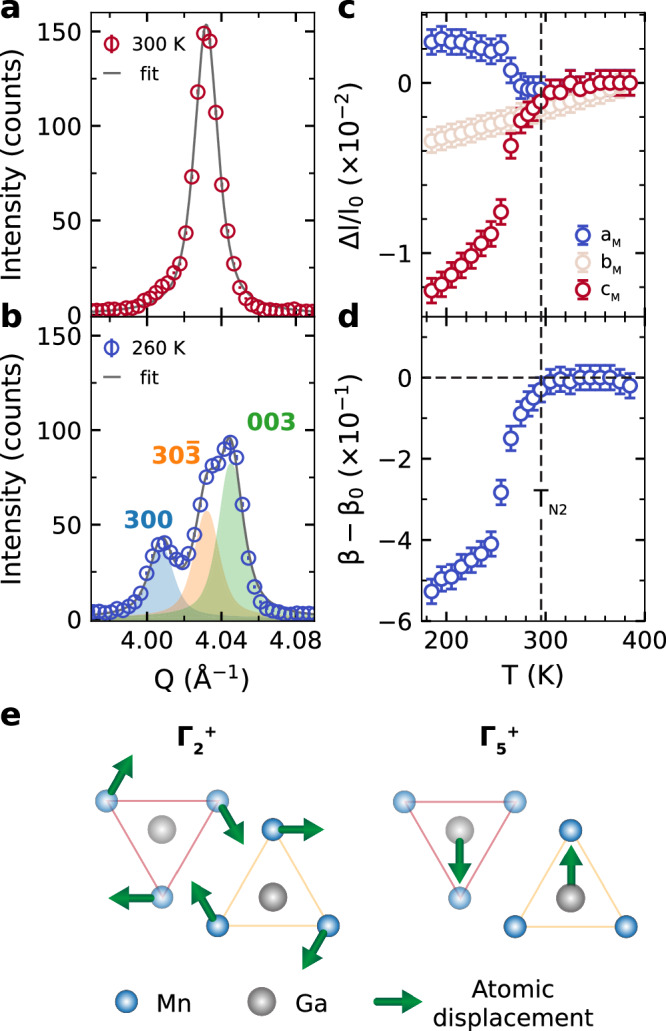
Structural phase transition at *T*_N2_ in Mn_3_Ga. **a**, **b** show hexagonal 300 Bragg peak and pseudo-Voigt function fits at 300 K and 260 K, respectively. The emergence of three distinct peaks below *T*_N2_ rules out an orthorhombic distortion and confirms a hexagonal-to-monoclinic structural transition. For the fit at 260 K, the full width at half maximum (FWHM) and mixing parameter (*η*) of the pseudo-Voigt function were fixed to the values obtained from the 300 K fit. **c**, **d** Temperature dependence of normalized refined lattice parameters and monoclinic cell angle *β*, extracted from Rietveld refinements using the monoclinic *P*2_1_/*m* (No. 11) space group against the neutron diffraction patterns. The results clearly show deviations from the high-temperature hexagonal symmetry below *T*_N2_. The normalized lattice parameters are defined as Δ*l*/*l*_0_ = [*l*(*T*) − *l*(*T*_0_)]/*l*(*T*_0_) (*l* = *a*_M_, *b*_M_, *c*_M_) to show the clear trend across *T*_N2_. **e** Two distortion modes (${\Gamma }_{2}^{+}$ and ${\Gamma }_{5}^{+}$) drive the structural transition from hexagonal *P*6_3_/*mmc* (No. 194) to monoclinic *P*2_1_/*m* (No. 11).

As shown in Fig. 2a, upon further cooling, high-resolution neutron diffraction reveals a clear splitting of Bragg peaks below *T*_N2_ = 295 K, indicating a symmetry-lowering distortion. High resolution neutron diffraction is critical to distinguish between the previously reported orthorhombic^22,24^ or monoclinic^23^ models for the low temperature structure. In the case of orthorhombic distortion, the hexagonal 300 peak is expected to split into two reflections (orthorhombic 330 and 060). In contrast, monoclinic symmetry results in three distinct peaks (monoclinic 300, 30$\bar{3}$ and 003) due to its lower symmetry and additional peak splitting. As shown in Fig. 3b, the observed peak splitting to three peaks at 260 K confirms the formation of a monoclinic unit cell below *T*_N2_. It is worth noting that the reflection from the MnO impurity phase near *Q* = 4 Å^−1^ is negligible and is expected to be less than 1% of the intensity of the hexagonal 300 reflection from Mn_3_Ga. We performed pseudo-Voigt function fits on the hexagonal 300 Bragg peak above and below *T*_N2_ (Fig. 3a, b). The full width at half maximum (FWHM) and mixing parameter (*η*) obtained from the fit at 300 K are used as constraints for fitting the 260 K data. Figure 3c, d shows the temperature dependence of lattice parameters. Clear splitting of the in-plane lattice parameters (*a*_M_ and *c*_M_) is observed, accompanied by a deviation of *β* from 120^∘^. The Rietveld analysis on the neutron diffraction data below *T*_N2_ reveals a space group *P*2_1_/*m* (No. 11), which shows a better refinement quality than the orthorhombic model *Cmcm* (No. 63) (see Supplementary Fig. 3). Symmetry analysis, based on the refined crystal structures and structural relationships determined using the BILBAO CRYSTALLOGRAPHIC SERVER^30–32^, reveals that two distortion modes, ${\Gamma }_{2}^{+}$ and ${\Gamma }_{5}^{+}$, contribute to this monoclinic distortion. These modes involve atomic displacements of both Mn and Ga restricted in the Kagome plane, as shown in Fig. 3e.

It is worth noting that our Mn_3_Ga sample shows a substantially higher MST temperature *T*_N2_ (295 K) compared with the 120 K^24^ and 170 K^22,23^ for samples with 70–74 at% Mn, and this value is slightly higher than the transition temperature of ≈285 K reported for Mn_2.95_Ga with the 74.7 at% Mn in a recent study^23^. This enhancement is likely attributed to the combined effects of improved crystallinity, variations in synthesis methods, and the stoichiometry of our sample. Indeed, as shown in Supplementary Fig. 1, changing the processing conditions of the Mn_3_Ga ingot used in this study can increase *T*_N2_ to near 320 K.

**Fig. 4:**
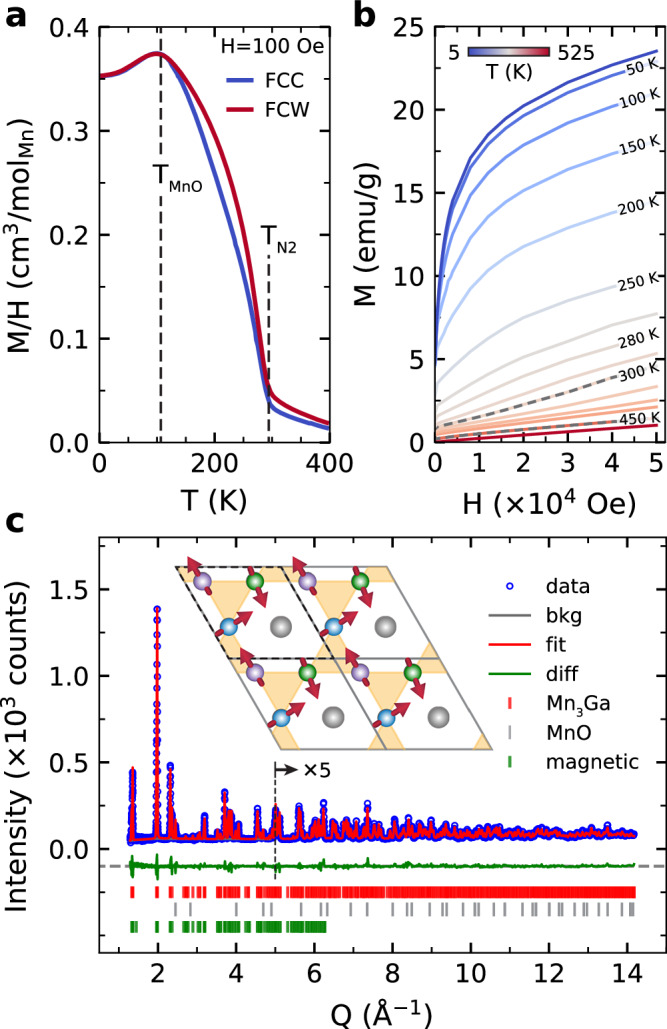
Magnetization and neutron diffraction results for *T* < *T*_N2_ in Mn_3_Ga. **a** Temperature-dependent magnetization measurements after field-cooled-cooling (FCC) and field-cooled-warming (FCW) protocols with *H* = 100 Oe show the onset of magnetic transition near *T*_N2_ = 295 K. The magnetization drops slightly near 110 K due to the magnetic transition of the MnO impurity phase. **b** Temperature-dependent *M*(*H*) curves measured from 5 to 525 K. **c** Rietveld refinement for neutron diffraction result at 30 K (*Rw* = 0.0769) using the $P{2}_{{1}^{{\prime} }}/{m}^{{\prime} }$ (BNS 11.54) magnetic space group.

Concurrent with this structural distortion, a new magnetic ground state emerges at *T*_N2_, as indicated by the onset of increasing magnetization upon cooling as shown in Fig. 4a and changes of the magnetic peak intensities (see Fig. 2a). The rapid increase of the magnetization and the pronounced deviation from linear *M*(*H*) behavior below *T*_N2_ in Fig. 4b indicate the emergence of a ferromagnetic component below *T*_N2_. Our neutron diffraction results show that the propagation vector remains zero (***k*** = 0). We found that neutron powder diffraction alone cannot unambiguously determine the magnetic structure due to overlapping nuclear and magnetic peaks and the subtlety of the simultaneous lowering of crystalline and magnetic symmetry. Only two magnetic models (A and H), shown in Supplementary Fig. 7, yielded excellent yet comparable refinement quality. To identify the correct magnetic structure, we combined the refinement results with our first-principles calculations including spin-orbit coupling (DFT+SOC). The DFT results validated magnetic model A since it exhibits the lowest energy among nine different magnetic orders in the monoclinic phase (see more details in Supplementary Information and Supplementary Figs. 6 and 7). The Rietveld refinement against the neutron diffraction data at 30 K using the magnetic model A ($P{2}_{{1}^{{\prime} }}/{m}^{{\prime} }$ (BNS 11.54)) is presented in Fig. 4c. The fitted atomic positions and magnetic moments are listed in Table 2. As shown in the inset of Figs. 4c and 1c, it is a canted AFM structure with substantial net moment (≈0.71 *μ*_B_ per Mn). The moments of Mn(1) and Mn(2) nearly cancel each other, and the net moment arises primarily from Mn(3), oriented nearly perpendicular to **a**_M_ in the monoclinic *a*_M_*c*_M_ plane, which elucidates the origin of the ferromagnetic component observed in the magnetization. The ordered moment per Mn site at 30 K is refined to be ≈2.56 *μ*_B_. Note that this magnetic structure differs significantly from that proposed in the previous report^23^. In addition, the decrease of the magnetization in Fig. 4a below ≈110 K is associated with the AFM transition of impurity phase MnO, as evidenced by the appearance of AFM peak $\frac{1}{2}\frac{1}{2}\frac{1}{2}$ of MnO below its Néel temperature *T*_MnO_ = 110 K (see Fig. 2a and Supplementary Fig. 4), consistent with previous reports^33,34^.

**Table 2:** Refined atomic positions and magnetic moments at 30 K

Atom	*x*	*y*	*z*	Symm. constr.	*M*_*x*_	*M*_*y*_	*M*_*z*_	∣M∣
Mn(1)	0.3433 (2)	0.2500	0.1766 (1)	*m*_*x*_, 0, *m*_*z*_	1.59 (16)	0.00	2.93 (29)	2.56 (24)
Mn(2)	0.8423 (5)	0.2500	0.1771 (9)	*m*_*x*_, 0, *m*_*z*_	2.54 (21)	0.00	−0.045 (18)	2.56 (28)
Mn(3)	0.8439 (5)	0.2500	0.6782 (1)	*m*_*x*_, 0, *m*_*z*_	−2.75 (23)	0.00	−0.43 (16)	2.56 (30)
Ga	0.3388 (4)	0.2500	0.6677 (6)	–	–	–	–	–

The refinement was performed in the magnetic space group $P{2}_{{1}^{{\prime} }}/{m}^{{\prime} }$ (BNS 11.54), with lattice parameters *a*_M_ = 5.4281(1) Å, *b*_M_ = 4.3396(1) Å, *c*_M_ = 5.3223(7) Å, and *β* = 119.45(7)°.

### Anomalous Hall effects and Weyl states above/below MST

**Fig. 5:**
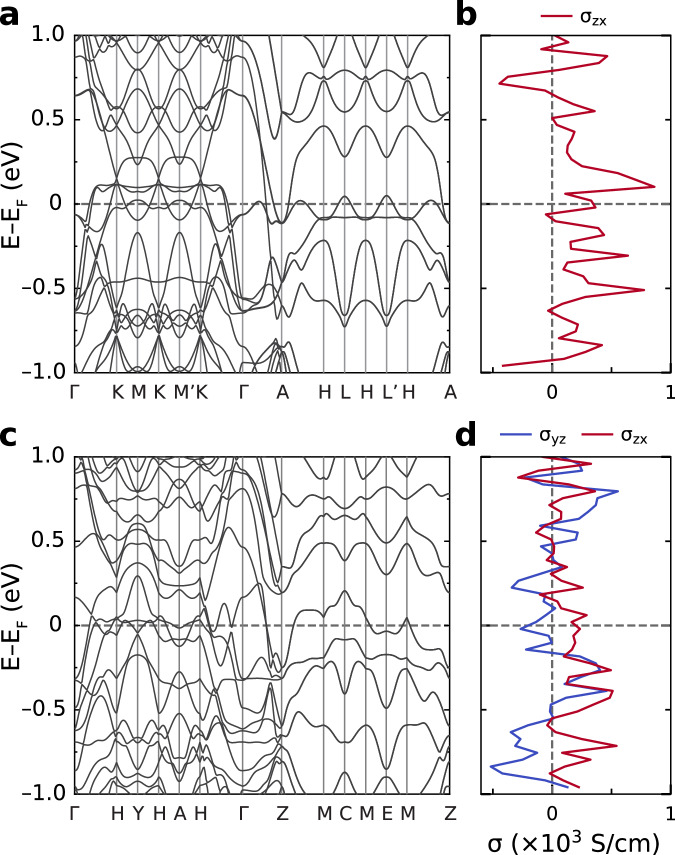
Electronic band structure and AHC from DFT calculations of Mn_3_Ga. **a**, **b** and **c**, **d** are for the hexagonal phase and the monoclinic phase, respectively. **a**, **c** show the band dispersions. We used the SeeK-path tool^74^ to determine high-symmetry **k** points, which are also indicated in Fig. 6a, c. **b**, **d** show the numerical results of AHC in the hexagonal phase and the monoclinic phase, respectively. Because of the high symmetry, only *σ*_*zx*_ is nonzero in the hexagonal phase, as shown in (**b**). In the monoclinic phase, there are obvious changes in AHC, with the emergence of nonzero *σ*_*yz*_ in (**d**).

With the established crystal and magnetic symmetries, we carried out first-principles calculations to locate potential Weyl nodes, determine their symmetry characteristics, and evaluate the associated anomalous Hall effect (AHE). The electronic band structure in the intermediate-temperature phase (*T*_N2_ < *T* < *T*_N1_) is shown in Fig. 5a. Since Mn_3_Ga shares the same symmetry as Mn_3_Sn and Mn_3_Ge above *T*_N2_, their band structures are correspondingly similar. However, reflecting the smaller number of total electrons, the Fermi level in Mn_3_Ga is lower by ~0.4 eV. This result is fully consistent with that in ref. ^29^ (Fig. 3a). Because of the spatial inversion symmetry (${{{\mathcal{I}}}}$) as well as the combined symmetry involving the mirror reflection with respect to a kagome plane and the time reversal (${{{{\mathcal{M}}}}}_{z}{{{\mathcal{T}}}}$), all bands on the *k*_*z*_ = 2*π*/*c* plane have twofold degeneracy (see A-H-L-H-L’-H-A line in Fig. 5a). The anomalous Hall conductivity for the hexagonal phase is shown in Fig. 5b. Mirror reflection symmetry about the *y* axis (${{{{\mathcal{M}}}}}_{y}$) forbids both *σ*_*xy*_ and *σ*_*yz*_, allowing only *σ*_*zx*_. The current *σ*_*zx*_ in the hexagonal phase is slightly different from the previous theoretical result^29^ due to minor variations in the lattice parameters, while the overall range remains consistent. The ${{{\mathcal{I}}}}$ and ${{{{\mathcal{M}}}}}_{z}{{{\mathcal{T}}}}$ symmetries also exist in the low-temperature monoclinic phase, leading to the twofold degeneracy in the dispersion relation on the *k*_*z*_ = 2*π*/*c* plane as shown in Fig. 5c (see Z-M-C-M-E-M-Z line). The corresponding AHC is shown in Fig. 5d. Although the ${{{{\mathcal{M}}}}}_{y}$ is broken in monoclinic symmetry, the combined symmetry ${{{{\mathcal{M}}}}}_{z}{{{\mathcal{T}}}}$ still remains, which prohibits only *σ*_*xy*_, allowing *σ*_*zx*_ and the emergence of non-zero *σ*_*yz*_.

**Fig. 6:**
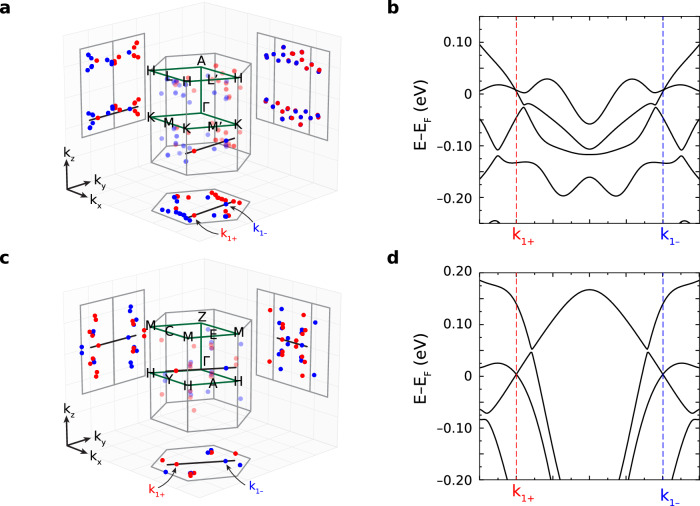
Weyl points and dispersions of Mn_3_Ga. **a**, **c** show Weyl points, which energy is within ±0.01 eV from *E*_F_. **b**, **d** show examples of band dispersions along momentum cuts indicated by black lines in **a** and **c** across Weyl points at **k**_1±_, showing type-II (**b**) and type-I (**d**) features in hexagonal and monoclinic phases, respectively.

Before going into the detailed analysis of Weyl points, we summarize the symmetry of Weyl points dictated by the magnetic and lattice symmetries of the system. For Mn_3_Sn and Mn_3_Ge with 120^∘^ ordering with the net magnetic moment along the *y* direction, there exist four important symmetries: a mirror reflection with respect to the (010) plane ${{{{\mathcal{M}}}}}_{y}$ and a half-lattice translation (*c*/2), a mirror reflection with respect to the (100) plane combined with the time reversal ${{{{\mathcal{M}}}}}_{x}{{{\mathcal{T}}}}$, a mirror reflection with respect to the (001) plane combined with the time reversal ${{{{\mathcal{M}}}}}_{z}{{{\mathcal{T}}}}$, as well the spatial inversion ${{{\mathcal{I}}}}$. When there is a Weyl point at (*k*_*x*_, *k*_*y*_, *k*_*z*_) with the chirality *χ*, because of ${{{{\mathcal{M}}}}}_{y}$, ${{{{\mathcal{M}}}}}_{x}{{{\mathcal{T}}}}$, and ${{{{\mathcal{M}}}}}_{z}{{{\mathcal{T}}}}$, there exist other Weyl points at (*k*_*x*_, −*k*_*y*_, *k*_*z*_), (*k*_*x*_, −*k*_*y*_, −*k*_*z*_), and (−*k*_*x*_, −*k*_*y*_, *k*_*z*_) with the opposite chirality −*χ*. Furthermore, ${{{\mathcal{I}}}}$ guarantees the existence of four additional Weyl points at (−*k*_*x*_, −*k*_*y*_, −*k*_*z*_) with the chirality −*χ*, as well as at (−*k*_*x*_, *k*_*y*_, −*k*_*z*_), (−*k*_*x*_, *k*_*y*_, *k*_*z*_), and (*k*_*x*_, *k*_*y*_, −*k*_*z*_) with the chirality *χ*. As shown in Fig. 6a, Weyl points in the high-temperature hexagonal phase of Mn_3_Ga, based on our calculations, clearly follow this rule.

In the low-temperature monoclinic phase of Mn_3_Ga, however, ${{{{\mathcal{M}}}}}_{y}$ and ${{{{\mathcal{M}}}}}_{x}{{{\mathcal{T}}}}$ symmetries are absent, and only ${{{{\mathcal{M}}}}}_{z}{{{\mathcal{T}}}}$ and ${{{\mathcal{I}}}}$ symmetries remain. As a consequence of this lower symmetry, Weyl points appear as quartets at (*k*_*x*_, *k*_*y*_, *k*_*z*_) and (*k*_*x*_, *k*_*y*_, −*k*_*z*_) with the chirality *χ* and (−*k*_*x*_, −*k*_*y*_, −*k*_*z*_) and (−*k*_*x*_, −*k*_*y*_, *k*_*z*_) with the chirality −*χ*, as shown in Fig. 6c. Therefore, our results suggest two distinct Weyl states with different AHEs below and above MST.

### Transport signatures of the topological phase transition

**Fig. 7:**
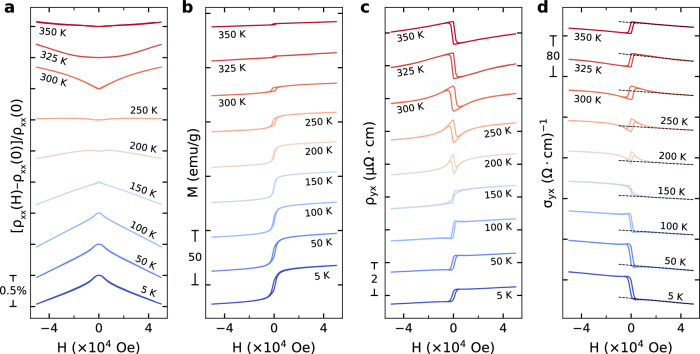
Evolution of transport and magnetic properties across *T*_N2_ in polycrystalline Mn_3_Ga. **a** Field-dependent longitudinal magneto-resistance (MR). **b** Magnetization *M*(*H*). **c** Hall resistivity *ρ*_*yx*_. **d** Hall conductivity *σ*_*xy*_(*H*). For visual clarity, curves at different temperatures are vertically offset by 0.5% in (**a**), 50 emu/g in (**b**), 2 μΩ cm in (**c**), and 80 Ω^−1^ cm^−1^ in (**d**).

To seek the experimental proof supporting our theoretically predicted topological Weyl state transition, we performed field-dependent transport measurements on longitudinal resistivity *ρ*_*xx*_ and total Hall resistivity *ρ*_*yx*_, as well as *M*(*H*) measurements across *T*_N2_. Figure 7a shows the longitudinal magnetoresistance, *MR* = [*ρ*_*xx*_(*H*) − *ρ*_*xx*_(0)]/*ρ*_*xx*_(0), which undergoes a distinct change in slope (∝*d*(*MR*)/*dH*) across the MST at *T*_N2_. At 350 K (*T*_N2_ < *T* < *T*_N1_), *d*(*MR*)/*dH* is positive and increases with magnetic fields in the 0–50 kOe region. However, upon cooling to 300 K near *T*_N2_, the positive *d*(*MR*)/*dH* becomes pronounced at low fields but decreases at higher fields. At lower temperatures, the positive *d*(*MR*)/*dH* evolves to a negative sign, and MR changes to negative over the whole measurement range. This sign change in MR was not observed in previously reported polycrystalline Mn_3_Ga samples^24,25^. Both the positive and negative MR from 350 K down to 5 K display nearly linear behavior without saturation, which is typical in Weyl semimetals^35–37^.

The temperature dependence of the magnetization *M*(*H*) curves is presented in Fig. 7b. In the *T*_N2_ < *T* < *T*_N1_ regime, the magnetization exhibits a weak magnetic hysteresis loop at low fields (<5 kOe) due to weak net component. At 350 K, we observe a small spontaneous magnetization of 0.008*μ*_*B*_ per Mn and a coercive field of  ≈1 kOe. At higher fields, *M*(*H*) shows a linear dependence on *H* and reaches a magnitude of only  ≈0.03*μ*_B_ per Mn at 50 kOe (350 K), suggesting that the nearly 120^∘^ chiral AFM order remains robust against the external field. Below *T*_N2_, the spontaneous magnetization increases sharply at low fields, indicating a significant net ferromagnetic component within the distorted AFM phase. While the magnetization continues to rise at higher fields, it only reaches approximately 0.25*μ*_B_ per Mn at 50 kOe (5 K), which is an order of magnitude lower than the ordered moment of 2.56*μ*_B_ per Mn determined by neutron diffraction. This discrepancy indicates that the applied field of 50 kOe is insufficient to fully polarize the distorted AFM order into a collinear ferromagnetic state.

The temperature dependence of the total Hall resistivity is shown in Fig. 7c. The total Hall resistivity^7,38^ is composed of the ordinary Hall resistivity (${\rho }_{yx}^{\,{{{\rm{N}}}}}$) and the anomalous Hall effect (${\rho }_{yx}^{\,{{{\rm{A}}}}}$), such that:

1 $${\rho }_{yx}={\rho }_{yx}^{\,{{{\rm{N}}}}}+{\rho }_{yx}^{{{{\rm{A}}}}}={R}_{0}H+{S}_{{{{\rm{A}}}}}{\rho }_{xx}^{2}M$$

where *R*_0_ is the ordinary Hall coefficient, *S*_A_ is a field-independent parameter, ${S}_{{{{\rm{A}}}}}{\rho }_{xx}^{2}$ is the conventional AHE coefficient proportional to magnetization. Note that the intrinsic ${\rho }_{yx}^{\,{{{\rm{A}}}}}$ reflects the integral of the Berry curvature over the entire Brillouin Zone for all occupied states.

**Fig. 8:**
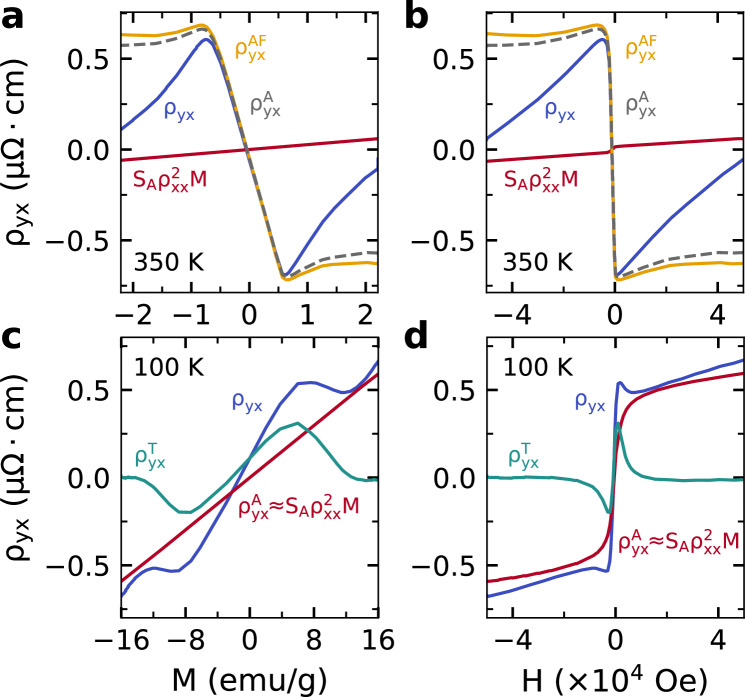
Decomposition of the Hall response at 350 and 100 K. Magnetization-dependent (**a**) and field-dependent (**b**) of total Hall resistivity *ρ*_*yx*_, and conventional AHE components (*S*_A_*ρ*_*xx*_*M*) and antiferromagnetic (${\rho }_{yx}^{\,{{{\rm{AF}}}}}$) contributions at 350 K. The dashed line indicates the total AHE component (${\rho }_{yx}^{\,{{{\rm{A}}}}}={S}_{{{{\rm{A}}}}}{\rho }_{xx}M+{\rho }_{yx}^{{{{\rm{AF}}}}}$). Magnetization-dependent (**c**) and field-dependent (**d**) of total Hall resistivity *ρ*_*yx*_, total AHE components (dominated by the conventional AHE, where ${\rho }_{yx}^{\,{{{\rm{A}}}}}\approx {S}_{{{{\rm{A}}}}}{\rho }_{xx}M$), and topological (${\rho }_{yx}^{T}$) contributions at 100 K.

At 350 K (*T*_N2_ < *T* < *T*_N1_), the AHE exhibits a negative (positive) sign for positive (negative) fields. A prominent sign reversal, accompanied by a distinct low-field hysteresis loop, is observed in *ρ*_*yx*_. As the field increases, the magnitude of the Hall signal decreases linearly. As further illustrated in Fig. 8a, the low-field nonlinearity and sign reversal of *ρ*_*yx*_ vs. *M* are attributed to an additional, unconventional AHE component, ${\rho }_{yx}^{\,{{{\rm{AF}}}}}$, which is intrinsically coupled to the nearly 120^∘^ triangular AFM order. The *R*_0_ and *S*_A_ coefficients are determined using the method described in previous studies on Mn_3_Sn^7^ and Mn_3_Ge^38^. We then isolate the individual ${S}_{{{{\rm{A}}}}}{\rho }_{xx}^{2}M$, ${\rho }_{yx}^{\,{{{\rm{AF}}}}}$, and ${\rho }_{yx}^{\,{{{\rm{A}}}}}={S}_{{{{\rm{A}}}}}{\rho }_{xx}^{2}M+{\rho }_{yx}^{{{{\rm{AF}}}}}$ components as a function of *M* and *H*, as shown in Fig. 8a, b, respectively. While the conventional AHE component ${S}_{{{{\rm{A}}}}}{\rho }_{xx}^{2}M$ is weak due to very low magnetization at 350 K and follows the standard proportionality to *M*, the much stronger ${\rho }_{yx}^{\,{{{\rm{AF}}}}}$ dominants ${\rho }_{yx}^{\,{{{\rm{A}}}}}$ and remains nearly independent of *M* or *H* in high field regions. Note that the *ρ*_*yx*_ profile of Mn_3_Ga at 350 K bears some similarity to the averaged Hall response based on the single crystal results on the isostructural Mn_3_Sn^7^ and Mn_3_Ge^15,38^, and that on polycrystalline Mn_3_Ga^25^.

The magnetic structure in this region involving a weak ferromagnetic component is characterized by the orthorhombic magnetic space group $C{m}^{{\prime} }c{m}^{{\prime} }$ in Fig. 1b, which explicitly breaks time-reversal symmetry, a fundamental requirement for the Weyl points and AHE. By using the hexagonal structure and $C{m}^{{\prime} }c{m}^{{\prime} }$ magnetic configuration, our DFT calculations have shown a robust AHE with *σ*_*yz*_ tensor driven by a significant enhancement of the Berry curvature localized around Weyl nodes near the Fermi level. The observed ${\rho }_{yx}^{\,{{{\rm{A}}}}}$, encompassing both conventional ${S}_{{{{\rm{A}}}}}{\rho }_{xx}^{2}M$ and unconventional ${\rho }_{yx}^{\,{{{\rm{AF}}}}}$, provides experimental evidence to support our theoretical predictions.

Upon cooling below *T*_N2_, *ρ*_*yx*_ shows a pronounced change in the nonlinearity, indicating a distinct shift in the AHE (${\rho }_{yx}^{\,{{{\rm{A}}}}}$) contribution that deviates from the linear field dependence characteristic of the ${\rho }_{yx}^{\,{{{\rm{N}}}}}$. This occurs near room temperature in our nearly stoichiometric Mn_3_Ga, significantly higher than the approximately 140 K observed in a previous report on Mn_3_Ga^25^. In addition, in sharp contrast to the high-temperature region, the *ρ*_*yx*_ becomes positive (negative) under positive (negative) fields at low temperatures, indicating a global sign reversal of the ${\rho }_{yx}^{\,{{{\rm{A}}}}}$. Notably, a pronounced hump-like feature emerges at moderate fields (<10 kOe) below ~150 K and vanishes at higher fields (see Fig. 8d and Supplementary Fig. 5c)—both of which are characteristic hallmarks of the topological Hall effect (THE)^39–41^. Although THE was previously reported in polycrystalline Mn_3_Ga^25^, it was associated with a different orthorhombic structure and occurred at a lower temperature, around 100 K.

Since the high-field region can be well described by ${R}_{0}H+{S}_{{{{\rm{A}}}}}{\rho }_{xx}^{2}M$, we determine the coefficients *R*_0_ and *S*_A_ by fitting the high-field data to the linear relation ${\rho }_{yx}/H={R}_{0}+{S}_{{{{\rm{A}}}}}({\rho }_{xx}^{2}M/H)$^25^ (see Supplementary Fig. 5d). The subtracted component ${\rho }_{yx}-{R}_{0}H-{S}_{{{{\rm{A}}}}}{\rho }_{xx}^{2}M$ is predominantly dominated by THE showing a clear hump at low fields that vanishes at high fields (see Fig. 8c, d). Consequently, the Hall resistivity at low temperatures is modeled as ${\rho }_{yx}={R}_{0}H+{\rho }_{yx}^{\,{{{\rm{A}}}}}+{\rho }_{yx}^{{{{\rm{T}}}}}\approx{R}_{0}H+{S}_{{{{\rm{A}}}}}{\rho }_{xx}^{2}M+{\rho }_{yx}^{{{{\rm{T}}}}}$ where the ${\rho }_{yx}^{\,{{{\rm{A}}}}}$ is well-described by the conventional AHE mechanism (${S}_{{{{\rm{A}}}}}{\rho }_{xx}^{2}M$). As shown in Fig. 8c, d, there is a dramatic increase in the ${S}_{{{{\rm{A}}}}}{\rho }_{xx}^{2}M$ component at 100 K compared to 350 K, which correlates with a significantly increased net moment. In addition to the emergence of a distinct ${\rho }_{yx}^{\,{{{\rm{T}}}}}$, the AHE (${\rho }_{yx}^{\,{{{\rm{A}}}}}$) at 100 K exhibits an opposite sign and different magnitudes from those at 350 K (see Fig. 8a, b).

To further demonstrate the topological contribution to the Hall response, we track the temperature evolution of the Hall conductivity, ${\sigma }_{xy}=-{\rho }_{yx}/{\rho }_{xx}^{2}$ (*ρ*_*xx*_ > > *ρ*_*yx*_, see Supplementary Fig. 5a). As shown in Fig. 7d, *σ*_*xy*_, which represents the average anomalous Hall conductivity of the three tensor components of the theoretical AHC, shows a significant change in nonlinearity and a sign reversal below *T*_N2_, which is attributed to the topological Weyl transition. In comparison to the maximum ${\sigma }_{xy}^{A}$ magnitude of 13.7 (Ωcm)^−1^ observed at 350 K (above *T*_N2_), the AHC increased significantly to 25.8 (Ωcm)^−1^ at 100 K (below *T*_N2_), as shown in Supplementary Fig. 5b. This behavior is consistent with the calculated enhancement of the AHC resulting from the appearance of an extra *σ*_*yz*_ tensor (Fig. 5d), which originates from the simultaneous structural and magnetic transformation at *T*_N2_. Therefore, our results on *ρ*_*yx*_ and *σ*_*xy*_ provide transport evidence that the MST reorganizes the Berry-curvature distribution and reconstructs the Weyl-node manifold in Mn_3_Ga.

## Discussion

### Unique mechanism of MST phase transition in Mn_3_X family

To gain deeper insight into the mechanisms driving phase transition in Mn_3_Ga, it is instructive to compare physical properties among those related compounds in the same family. All three materials, Mn_3_*X* (*X* = Ga, Ge, Sn), can be stabilized in the hexagonal *D*0_19_ structure (Fig. 1a) and their magnetic ordering breaks time-reversal symmetry^15,23,25,42,43^. Both theoretical and experimental studies have shown that the chiral antiferromagnetic order in Mn_3_Sn, Mn_3_Ge, and Mn_3_Ga gives rise to a pronounced anomalous Hall effect (AHE) and spin Hall effect (SHE), driven by Berry curvature associated with their magnetic order^29^. However, Mn_3_Ga is distinguished from Mn_3_Sn and Mn_3_Ge by its electronic configuration, possessing one fewer valence electron, which leads to two key consequences.

First, while the electronic band structures of Mn_3_*X* (*X* = Ga, Ge, Sn) are qualitatively similar, differences in valence electron count significantly impact their topological behavior. In Mn_3_Ge, the Fermi level resides close to several Weyl nodes, resulting in strong Berry curvature and a large intrinsic AHE that persists up to high temperatures^15^. Mn_3_Sn exhibits stronger spin-orbit coupling, which leads to partial annihilation and displacements of Weyl nodes slightly away from the Fermi level^7^. By contrast, the reduced electron count in Mn_3_Ga lowered the Fermi energy, suppressing the net Berry curvature at the Fermi surface and weakening the AHE, while potentially enhancing the spin Hall conductivity through redistribution of the Berry curvature proposed by a previous study^29^.

Second, while the hexagonal structure in Mn_3_Ge and Mn_3_Sn persists down to the lowest temperature, the presence of a magnetostructural phase transition in Mn_3_Ga suggests that orbital degeneracy is lifted^23,25^. This indicates a strong coupling between lattice, orbital, and spin degrees of freedom in Mn_3_Ga, which is absent in Mn_3_Ge and Mn_3_Sn. What is the driving force for the magnetostructural phase transition at *T*_N2_ in Mn_3_Ga? According to our DFT calculation, the total energy of the most stable magnetic structure in the monoclinic phase is −30.043 eV f.u.^−1^, while that of the 120^∘^ triangular AFM structure in the hexagonal phase is −30.002 eV f.u.^−1^ (both are computed at *T* = 0). The magnetostructural phase transition between the two phases might be reasonably accounted for by thermal effects, including the reduction in the ordered moment and phonon excitations, that could overcome the small energy difference 0.041 eV ~ 475 K. The unique combination of monoclinic structure and the canted AFM order with net ferromagnetic component in Mn_3_Ga leads to a redistribution of Weyl nodes and drives a topological Weyl phase transition, which was not observed in Mn_3_Ge and Mn_3_Sn.

### Emergent anomalous phase at low temperatures and fields

The emergence of the THE is rooted in the scalar spin chirality of non-coplanar magnetic textures and acts as a source of real-space Berry phase^40,44^. While neutron diffraction at zero field reveals a distorted AFM order with monoclinic magnetic symmetry, this configuration remains coplanar. Consequently, the scalar spin chirality, defined by **S**_*i*_ ⋅ (**S**_*j*_ × **S**_*k*_)^40,44^ for a spin triad, is restricted to zero. The observation of a THE at low fields ( < 10 kOe) suggests that the ground-state distorted AFM order undergoes a field-induced transition to a non-coplanar state with non-zero spin chirality. A plausible scenario is that the applied field induces spin canting along the out-of-plane *c*_H_ axis. Theoretical calculations have demonstrated that such out-of-plane canting can generate substantial Berry curvature along nodal lines in *k*-space, enhancing the AHE and potentially fostering the formation of new Weyl points, as seen in the hexagonal phase of Mn_3_Sn^44^. Thus, while the THE is fundamentally a real-space Berry phase phenomenon, the underlying spin canting simultaneously modifies the *k*-space Berry curvature. The coexistence of AHE and THE in Mn_3_Ga therefore represents a profound convergence of real-space and momentum-space topological effects.

In contrast to the high-temperature nearly 120^∘^ triangular AFM phase, which exhibits no THE up to 50 kOe, the THE becomes prominent below ≈150 K. This temperature corresponds to the regime where the rate of increase in magnetization begins to diminish upon cooling (Supplementary Fig. 1). Furthermore, the THE coincides with the rapid low-field increase in magnetization observed in *M*(*H*) curves below 10 kOe (Fig. 7b). These correlations indicate that the response of the net FM component to the external fields may be closely related the spin chirality and the appearance of THE in Mn_3_Ga. At higher fields exceeding ≈10 kOe, the *M*(*H*) curves exhibit a continuous increase with reduced *dM*(*H*)/*dH*, reflecting the response of the primary AFM component to the field. The disappearance of the THE in this regime suggests that the spin chirality reverts to zero. Furthermore, the THE is often considered one hallmark of the skyrmion phase^40^, like the A phase in MnSi^45^. Thus, an anomalous phase with spin chirality exists at low fields below approximately 150 K in Mn_3_Ga. Our results may motivate further investigation into the field-dependent magnetic structures/spin chirality, topological states, and THE-related phenomena in Mn_3_Ga.

### MST-driven topological Weyl phase transition

First-principles calculations including spin-orbit coupling demonstrate that both the hexagonal and monoclinic phases of Mn_3_Ga host symmetry-protected Weyl nodes, though with fundamentally different distributions governed by crystal and magnetic symmetries. In the hexagonal phase (*T*_N2_ < *T* < *T*_N1_), the magnetic structure we observe is nearly 120^∘^ chiral AFM order with a weak in-plane FM component, consistent with the theoretical model reported previously^23^. The combination of mirror, time-reversal, and inversion symmetries produces a high multiplicity of Weyl nodes of Mn_3_Ga arranged in symmetry-related sets, generating a Berry curvature landscape similar to that predicted for Mn_3_Sn and Mn_3_Ge with many pairs of type-II Weyl nodes identified^17,46,47^. Figure 6b shows examples of tilted type-II Weyl nodes in the hexagonal phase for Mn_3_Ga. Our calculations demonstrate that the integration of the Berry curvature over the entire Brillouin Zone for all occupied states, including the region around the Weyl points in this band structure, gives rise to a substantial *σ*_*zx*_ component of the AHE (Fig. 5b). This theoretical result is corroborated by our experimental observations of the AHE, which encompass both the conventional ferromagnet-like contribution and an unconventional AHE component (Figs. 7c, d and 8a, b).

In *T* < *T*_N2_, our study resolves the previously debated crystal and magnetic structures. This newly identified spin configuration with a strongly distorted AFM order, combined with the symmetry-lowering structural distortion below *T*_N2_, breaks ${{{{\mathcal{M}}}}}_{y}$ and ${{{{\mathcal{M}}}}}_{x}{{{\mathcal{T}}}}$ symmetries while preserving ${{{{\mathcal{M}}}}}_{z}{{{\mathcal{T}}}}$ and inversion (${{{\mathcal{I}}}}$). This symmetry reduction reorganizes the Weyl node network with redistributed momentum-space positions and alters chirality arrangements, as shown in Fig. 6. Notably, the symmetry-driven reconfiguration of the Weyl nodes could be accompanied by the change in the type of Weyl points from strongly tilted type-II character^47^ in the hexagonal phase (Fig. 6b) to the upright type-I character^47^ in the monoclinic phase (Fig. 6d), representing a rare example of a temperature-dependent intrinsic Weyl phase transition. This transition is characterized by a modification of the AHC tensor, specifically the emergence of an additional *σ*_*yz*_ component alongside *σ*_*zx*_, which leads to an enhancement of the total AHC, as shown in Fig. 5d. Experimentally, we observed a reversal in the sign of the AHC and a twofold increase in its maximum magnitude at low temperatures, as shown in Fig. 7d and Supplementary Fig. 5b, compared to the profiles observed at 350 K in the hexagonal phase. Our theoretical results showed that the Berry curvature distribution and the Weyl-node topology in the monoclinic phase are distinct from those in the hexagonal phase, leading to the observed changes in the anomalous Hall effects above/below *T*_N2_. Furthermore, the exclusive appearance of the THE in the monoclinic phase suggests that the Weyl state, as well as the interplay between momentum-space and real-space Berry curvatures, can be effectively tuned by magnetic fields. The topological reconstruction, driven by a magnetostructural transition, demonstrates how the concurrent breaking of crystal and magnetic symmetries can reshape the electronic structure and generate a novel topological Weyl state and THE.

### Engineering magnetostructural transitions

Our results have demonstrated that magnetostructural phase transitions play a decisive role in governing the topological properties of Mn_3_Ga. MSTs are known to occur across a broad class of materials, such as Heusler Ni-Mn-*X* (*X* = Ga, Sn) alloys^48,49^, FeRh intermetallic^50,51^, MnAs compound^52^, perovskite manganites^53^ and spinel oxide MnV_2_O_4_^54^. Interestingly, MSTs can be triggered through targeted chemical design even in cases where MST is not present in the parent compounds^55–57^. For example, the parent compound MnCoGe lacks magnetostructural coupling, exhibiting a substantial temperature separation of around 300 K between its magnetic transition temperature (*T*_C_) and structural transition temperature (*T*_S_). However, introducing only a few percent of interstitial boron can tune the magnetic and structural transitions to coincide for inducing the occurrence of MST^55^. In another example of the MnNiSi-CoNiGe alloy, co-substitution of Co and Ge significantly shifts the structural transition temperature, and aligns it with the magnetic ordering temperature, thereby driving the MST and enhancing magneto-responsive performance^57^. Furthermore, the MSTs can be controlled and tuned effectively through chemical substitution^56,58^, heat treatment^59,60^, magnetic field^61^, pressure^62^, or film engineering^63^. Therefore, investigating magnetostructural transitions opens broad possibilities for realizing topological quantum phases governed by simultaneously broken crystal and magnetic symmetries.

In summary, we report Mn_3_Ga as a rare system that undergoes an intrinsic topological Weyl phase transition between two distinct Weyl states near room temperature, driven by a magnetostructural transition. The magnetostructural transition involves a symmetry-lowering lattice distortion and a magnetic structural transformation, leading to a Weyl phase transition from a primary type-II Weyl state to a distinct Weyl state, and a pronounced change of both signs and magnitudes of the anomalous Hall effect, as well as the emergence of the topological Hall effect. Associated with the THE, an anomalous phase with spin chirality is identified at low fields (<10 kOe) below approximately 150 K. Our findings reveal a compelling route to alternate emergent topological states through intrinsic magnetostructural transition in correlated quantum materials. Further studies on Mn_3_Ga using angle-resolved photoemission spectroscopy, scanning tunneling microscopy, AHE measurements on single crystals, as well as field tuning and film engineering, are required to bring up further new physics and potential applications through the controlled switching and manipulation of two distinct Weyl states. Our study should motivate future theoretical and experimental studies on other materials in which the magnetostructural transition occurs or can be induced for identifying and realizing novel topological Weyl states.

## Methods

### Sample preparation

High-purity manganese (99.95%) and gallium (99.9999%) were combined in a 3:1 molar ratio and arc-melted to form an initial alloy. The resulting ingot was sealed in an evacuated fused silica ampoule and annealed at 673 K for 10 days, resulting in the formation of Mn_3_Ga in the tetragonal phase. To obtain the hexagonal phase, the ingot was resealed under vacuum, heated to 873 K for 2 h, and subsequently quenched in room-temperature water.

Further preparation for the powder neutron diffraction sample involved grinding part of the ingot into powder using a percussion mortar and pestle, followed by ball milling in a tungsten carbide-lined crucible with tungsten carbide balls. The powder was annealed (873 K) for 17 h and quenched in room-temperature water, resulting in well-crystallized hexagonal Mn_3_Ga powder.

To investigate the impact of ball milling on the magnetic transition temperature (*T*_N2_), a separate bulk fragment was cut from the ingot and annealed directly at 873 K for 17 h without prior milling. Magnetization measurements revealed an increased transition temperature of *T*_N2_ ≈ 320 K, as shown in Supplementary Fig. 1.

Further preparation for the electrical transport measurements involved cutting and grinding part of the ingot into rectangular parallelepiped samples. To relieve surface strain and damage induced during the shaping process, these samples underwent an initial annealing at 873 K for 17 h followed by a water quench. A subsequent annealing step was performed at 873 K for 6 h. The resulting *T*_N2_ was approximately 310 K, close to the transition temperature observed in the powder samples used for neutron diffraction.

### Neutron diffraction

Neutron powder diffraction measurements were carried out using the high-resolution neutron diffractometer POWGEN at the Spallation Neutron Source (SNS), Oak Ridge National Laboratory. A 3.5 g powder sample of Mn_3_Ga was loaded into a vanadium can, which was subsequently mounted in a cryofurnace (JANIS) to cover temperature region from 30 K to 600 K. An orange cryostat was also used for complementary data collection from 2 K to 300 K. Isothermal neutron diffraction measurements were conducted at 30, 200, 350, and 600 K using the neutron frame 1 (center wavelength: 0.8 Å) to cover wide *Q* region from 1 to 14 Å^−1^. To specially check the small distortion, the high-resolution setup with neutron frame 2 (center wavelength: 1.5 Å) and neutron frame 3 (center wavelength: 2.665 Å) was also used for the measurements. At each measured temperature, sufficient waiting time was allowed to ensure thermal stability. Continuous temperature-dependent measurements from 30 to 600 K were conducted using a cryofurnace (JANIS) to capture the evolution of structural and magnetic Bragg peaks across two phase transitions.

Neutron powder diffraction data were used to refine the crystal and magnetic structures of Mn_3_Ga using the GSAS-II software package^64^. ∣*M*∣ represents the size of the ordered magnetic moment vector **M** = (*m*_*x*_, *m*_*y*_, *m*_*z*_). It was calculated based on the (*m*_*x*_, *m*_*y*_, 0) components for the hexagonal phase or the (*m*_*x*_, 0, *m*_*z*_) components for the monoclinic phase, incorporating the respective inter-component angles. The GSAS-II software computed these values directly, which were subsequently extracted from the refinement output (.lst) files. To obtain the average net moment per Mn, the vector sum of all Mn moments in the magnetic unit cell was normalized by the six Mn atoms contained within one magnetic unit cell. The BILBAO CRYSTALLOGRAPHIC SERVER^30–32^ and ISODISTORT^65^ were used to determine the structural distortion modes and symmetry-allowed magnetic space groups.

### Magnetic characterization and transport measurements

Magnetization measurements were performed using a Magnetic Property Measurement System (MPMS-XL) from Quantum Design. For measurements between 5 and 395 K, 46 mg of powder sample was loaded into gelcaps and mounted into a plastic drinking straw. For measurements between 300 and 550 K, 30 mg of the powder sample was loaded in a fused silica tube.

Electrical transport measurements were carried out in a Quantum Design Physical Property Measurement System (PPMS) and Quantum Design Dynacool using the Electrical Transport Option (ETO). Two samples were measured and showed consistent results. Magnetic field-dependent measurements in the longitudinal resistivity (*ρ*_*xx*_) and Hall (*ρ*_*yx*_) configurations were collected under isothermal conditions by stepping the applied field from 50 kOe to −50 kOe, then back to 50 kOe. For *ρ*_*xx*_, data from a sample with leads attached in a longitudinal configuration were symmetrized by averaging the results from the decreasing-field scan and the increasing-field scan (to remove Hall contributions arising from lead misalignment). For *ρ*_*yx*_, data from a sample with leads attached in a transverse configuration were antisymmetrized by taking one half of the difference between the resistance measured on decreasing and on increasing the field (to remove longitudinal contributions arising from lead misalignment). Note that in Fig. 7 full loops of symmetrized and antisymmetrized transport data are shown. Since they were determined from a single loop of raw data, the branches in the full loops of processed data are related and do not represent independent data collected on increasing and decreasing the field. Corresponding isothermal magnetization curves were measured on a piece cut from the Mn_3_Ga ingot and heat-treated in the same way as the transport samples.

### Density functional theory

To gain insight into the magnetic ordering and potential Weyl physics in the regimes *T*_N2_ < *T* < *T*_N1_ and *T* < *T*_N2_, we carried out DFT calculations for Mn_3_Ga using the hexagonal and monoclinic structures obtained from neutron diffraction measurements at 350 K and 30 K, respectively. The projector augmented wave method^66,67^ is used with the generalized gradient approximation in the parametrization of Perdew, Burke, and Enzerhof^68^ for exchange-correlation as implemented in the VIENNA *ab* INITIO SIMULATION PACKAGE (VASP)^69,70^. For Mn, we used a potential in which the *p* states are treated as valence states (Mn_*pv*_ in the VASP distribution), and for Ga, a potential in which *d* states are treated as valence states (Ga_*d*_). For the electronic self-consistent calculations, we use a 12 × 12 × 12 **k**-point grid and an energy cutoff of 500 eV. Spin-orbit coupling (SOC) is included, whereas the  + *U* correction is omitted because Mn_3_Ga is an itinerant magnetic system. To investigate the ground-state magnetic structure, we consider nine initial spin configurations that conform to the lattice symmetry (see supplementary Fig. 6) and let them relax to stable configurations, as displayed in supplementary Fig. 7.

Subsequently, the topological properties of Mn_3_Ga are investigated using an effective tight-binding model. We employed the WANNIER90 code^71^ to derive the maximally localized Wannier functions for both the hexagonal and monoclinic phases of Mn_3_Ga. The effective tight-binding models were refined utilizing the WANNSYMM package^72^. The locations and chiralities of the Weyl points were then analyzed using these effective tight-binding models with the WANNIERTOOLS package^73^. For the calculations on AHC, we used a fixed laboratory frame with **x**∥**a**_H_∥**c**_M_, **z**∥**c**_H_∥**b**_M_ and **y** is set by the right-hand rule (gray axes in Fig. 1b).

## Supplementary information

Supplementary Information

Peer Review File

## Source data

Source Data

## Data Availability

The datasets generated and analyzed during this study are available in the Zenodo repository at 10.5281/zenodo.18928689. The repository includes neutron diffraction, magnetization, and transport data, as well as density functional theory (DFT) calculation results associated with this work. Source data are provided with this paper.

## Code availability

The computer code supporting the findings of this study is available from the corresponding author upon reasonable request.
