# Supplementary material for: Intrinsic topological Weyl phase transition induced by a magnetostructural transformation in a kagome magnet
Source: Nat Commun. 2026 Apr 11;17:5063. doi: 10.1038/s41467-026-71683-7 (PMC13243550; doi:10.1038/s41467-026-71683-7)
Supplement: Supplementary file 1 — Supplementary Information [file 41467_2026_71683_MOESM1_ESM.pdf]

Supplementary Information for Intrinsic Topological Weyl Phase Transition Induced by a Magnetostructural Transformation in a Kagome Magnet

Tsung-Han Yang[1], Satoshi Okamoto[2,*], D. Alan Tennant[3,4,5], Michael A. McGuire[2,*], Qiang Zhang[1,*]

[1] Neutron Scattering Division, Oak Ridge National Laboratory, Oak Ridge, Tennessee 37831, USA

[2] Materials Science & Technology Division, Oak Ridge National Laboratory, Oak Ridge, Tennessee 37831, USA

[3] Department of Physics & Astronomy, University of Tennessee, Knoxville, TN, USA

[4] Shull Wollan Center, Oak Ridge National Laboratory, Oak Ridge, TN 37831, USA

[5] Department of Materials Science and Engineering, University of Tennessee, Knoxville, TN, USA

* Corresponding author: okapon@ornl.gov

* Corresponding author: mcguirema@ornl.gov

* Corresponding author: zhangq6@ornl.gov

DFT of magnetic structures in the monoclinic phase

Here, we provide the theoretical detail of the investigation of the ground-state magnetic ordering with the low-temperature monoclinic structure. As described in the main text, we carry out density functional theory (DFT) calculations. We consider nine initial spin configurations, that are consistent with the lattice symmetry. These configurations are grouped as shown in Fig. 6: the first three and second three orderings exhibit 120°-like spin arrangements but with opposite chirality, and the last three orderings feature nearly-collinear arrangements. We allowed these initial states to relax to stable configurations. The resulting magnetic orderings are summarized in Fig. 7 sorted by the ascending order of total energy. The same labels (A-I) used for the initial states were retained. The lowest-energy orderings have the same spin chirality as that with high-temperature hexagonal structure as well as that in Mn_3Sn and Mn_3Ge, but their spin orientations are modified due to the lower crystal symmetry. The magnetic ordering labeled **A** is consistent with our neutron diffraction measurements.

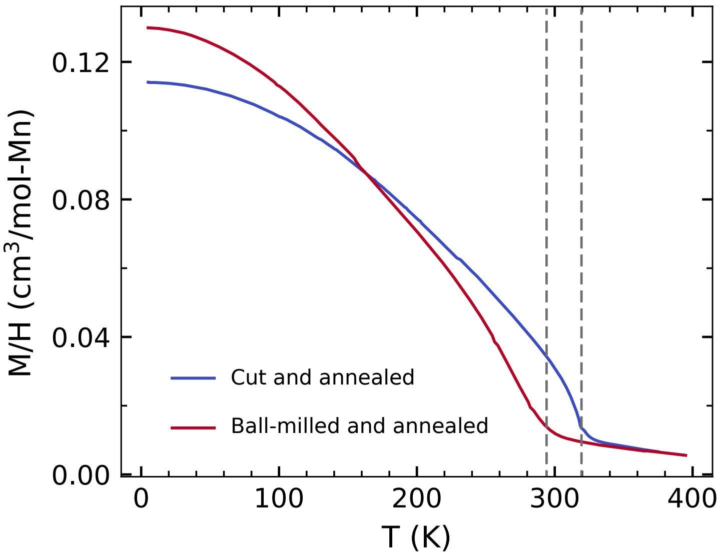

Supplementary Figure 1: **Ball milling effect on the locations of $T_{\mathbf{N2}}$.** Both originated from the same cast and annealed boule of Mn_3Ga. The red curve represents the sample that was ball milled and annealed for 17 hours (T_{N2} = 295 K). The blue curve represents the sample that was cut from the boule, and annealed directly for 17 hours without prior ball milling process (T_{N2} = 320 K). The data were measured in an applied field of 10 kOe.

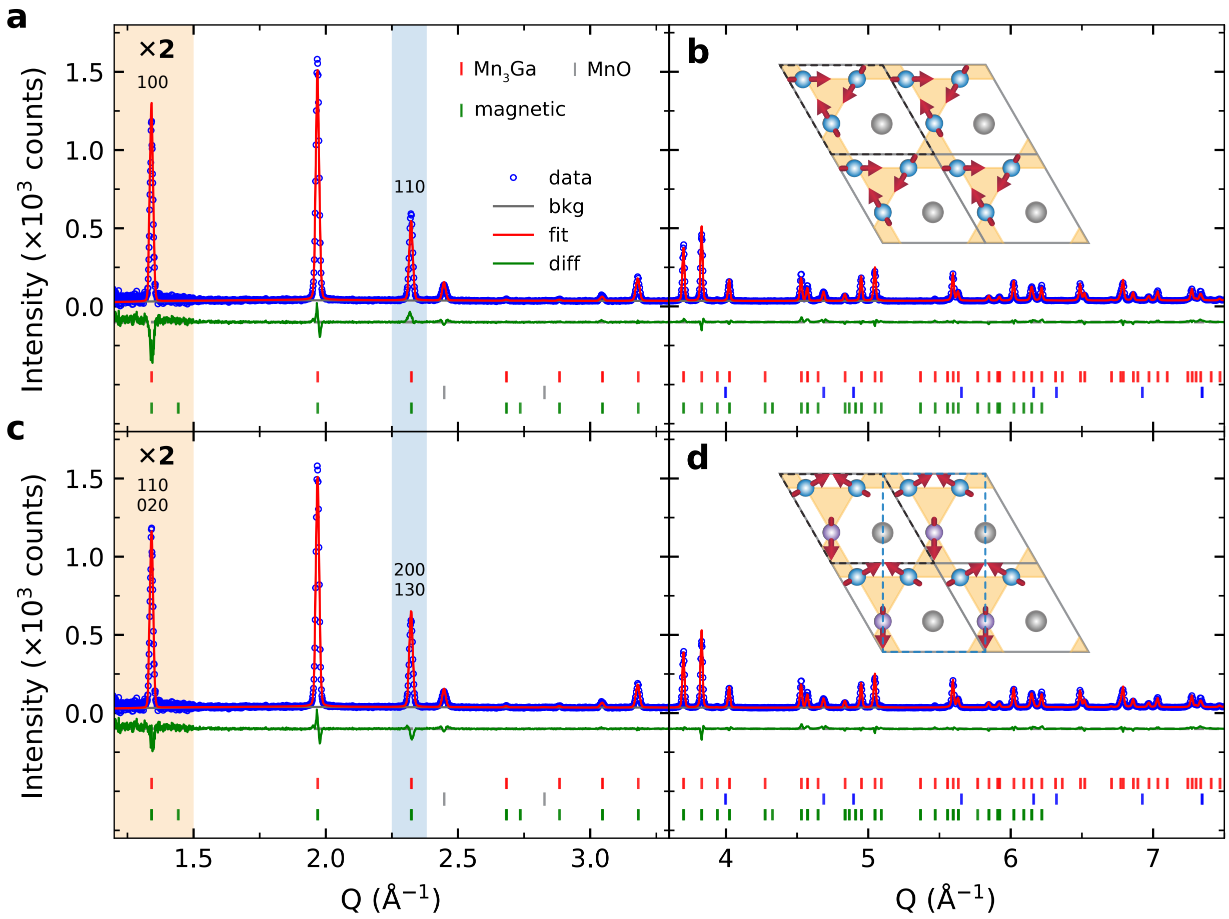

Supplementary Figure 2: **Rietveld refinements of neutron powder diffraction patterns at 350 K. a**, **b** Fits obtained using the hexagonal magnetic model ($P6_{3'}/m'mc'$ (BNS 194.269)) and **c**, **d** the orthorhombic magnetic model ($Cm'cm'$ (BNS 63.464)). Low–Q regions **a**, **c** highlight the magnetic scattering, where the orthorhombic model provides a substantially improved refinement of the data and peak ratio $I(100)_H/I(110)_H$, as emphasized in the shaded regions. The yellow shaded areas are magnified by a factor of 2 for clarity. High–Q regions **b**, **d** show comparable agreement for both models, indicating an equivalent description of the nuclear lattice contribution.

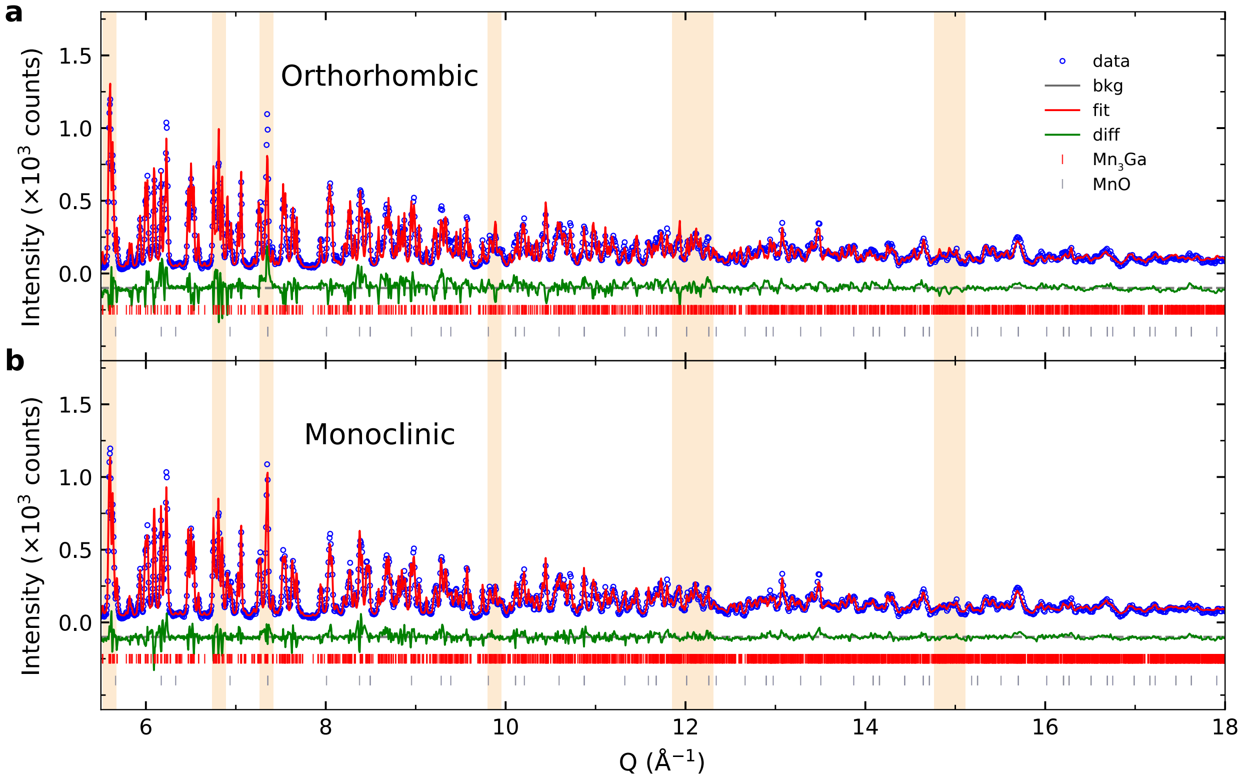

Supplementary Figure 3: **Rietveld refinements of high-Q neutron powder diffraction data with negligible magnetic contribution at 200 K. a** Orthorhombic ($Cmcm$) and **b** monoclinic ($P2_1/m$) structural models. The monoclinic model provides a better fit ($R_w = 0.0716$) than the orthorhombic model ($R_w = 0.0915$), particularly in reproducing the shapes and intensities of Bragg peaks. Shaded regions indicate areas where the monoclinic model shows noticeable improved agreement.

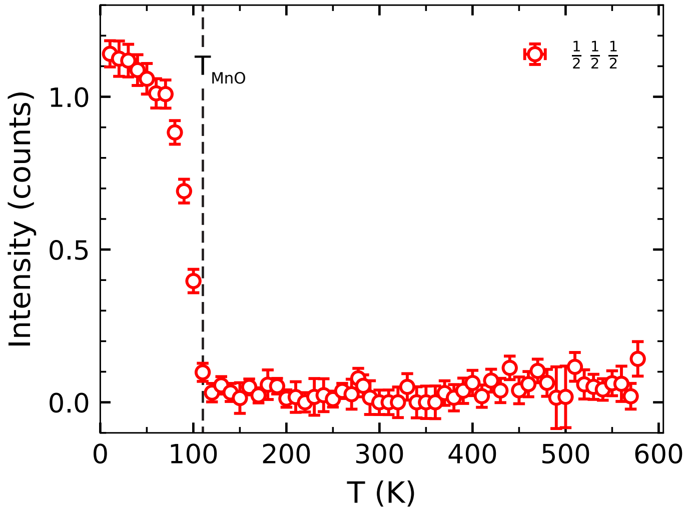

Supplementary Figure 4: **Temperature dependence of the $\frac{1}{2}$ $\frac{1}{2}$ $\frac{1}{2}$ magnetic Bragg peak intensity in MnO.** The peak intensity was extracted by fitting with a pseudo-Voigt function using a fixed mixing factor and full width at half maximum for each temperature. The magnetic transition temperature near 110 K is consistent with previous reports.

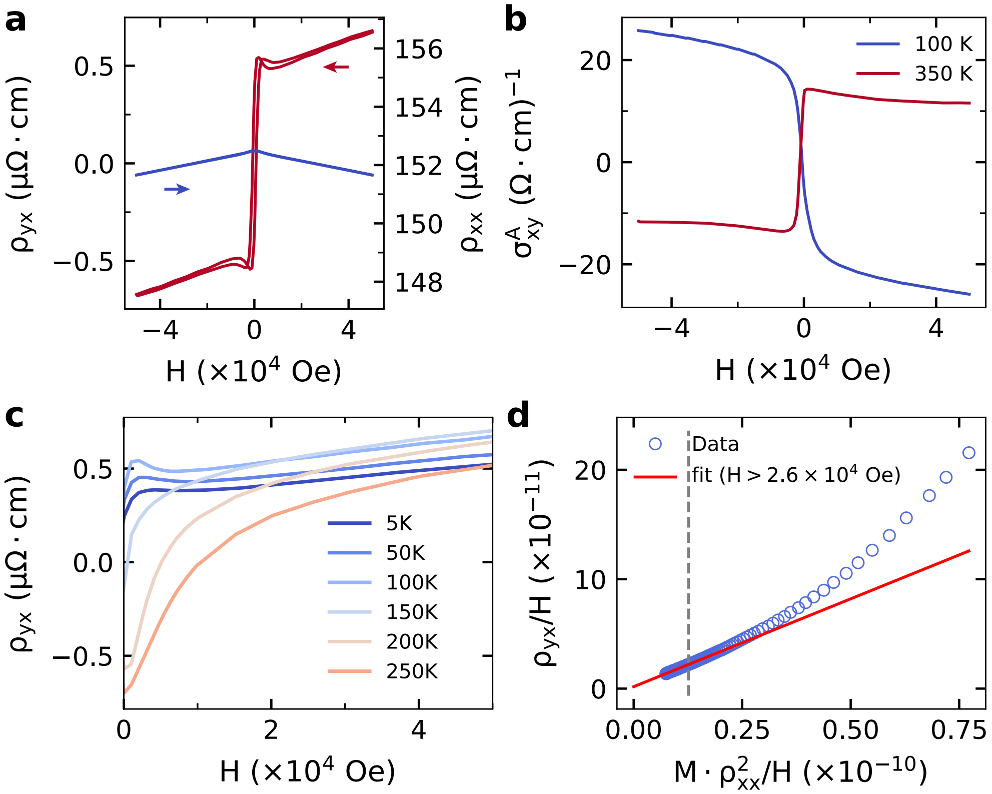

Supplementary Figure 5: **Field-dependent Hall resistivity ρ_{yx}, longitudinal resistivity ρ_{xx}, and anomalous Hall conductivity σ_{xy}^{A} component.** **a** Field dependence of $\rho_{yx}(H)$ and $\rho_{xx}(H)$ at 100 K. The longitudinal resistivity is significantly larger than the Hall resistivity over the entire field range. **b** Anomalous Hall conductivity at 100 K and 350 K. **c** Expanded view of $\rho_{yx}(H)$ in the vicinity of T_{N2}, highlighting the evolution across the transition. **d** Scaling analysis used to extract the ordinary Hall effect (OHE) and anomalous Hall effect (AHE) contributions.

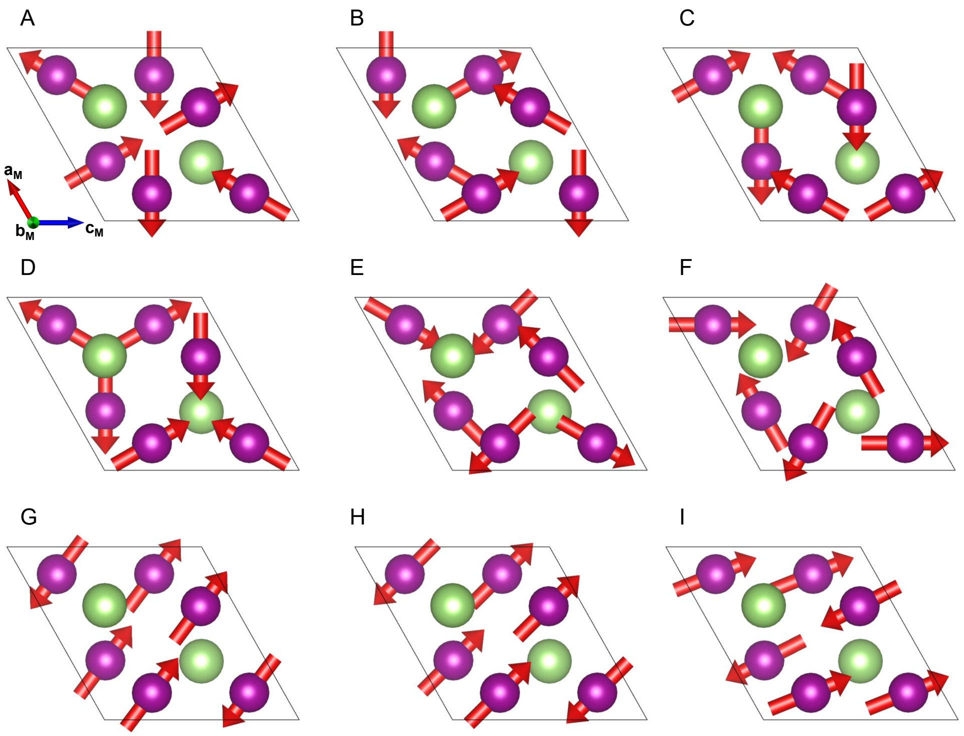

Supplementary Figure 6: **Initial magnetic configurations used in DFT calculation for the low-temperature monoclinic structure.** Magnetic configurations (A–C) and (D–F) have 120°-like spin arrangements with opposite chirality, while (G–I) have nearly-collinear arrangements.

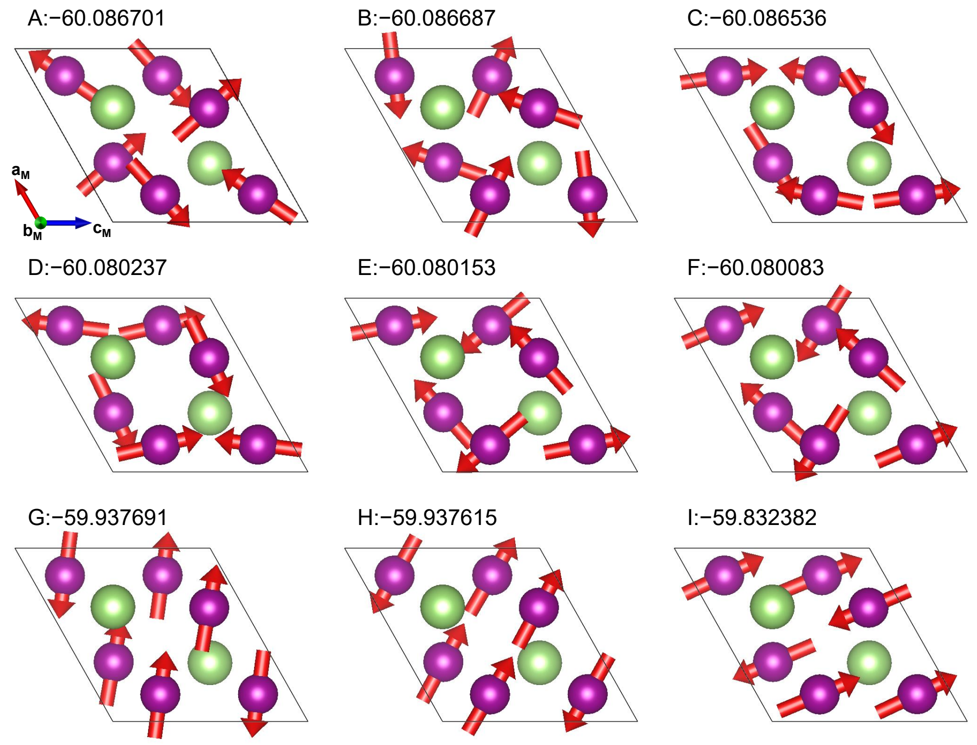

Supplementary Figure 7: **DFT calculation results of the total energy (in eV per unit cell), arranged in ascending order from A to I, for nine magnetic configurations of the low-temperature monoclinic structure.** (A–I) denote the nine stable magnetic configurations obtained after relaxation from the corresponding initial configurations shown in Supplementary Figure 6. The magnetic configuration designated as A aligns with the results obtained from the Rietveld analysis of neutron diffraction data.
