# Supplementary material for: Intrinsic topological Weyl phase transition induced by a magnetostructural transformation in a kagome magnet
Source: Nat Commun. 2026 Apr 11;17:5063. doi: 10.1038/s41467-026-71683-7 (PMC13243550; doi:10.1038/s41467-026-71683-7)
Supplement: Supplementary file 2 — Peer Review File [file 41467_2026_71683_MOESM2_ESM.pdf]

nature portfolio

Peer Review File

Intrinsic Topological Weyl Phase Transition Induced by a Magnetostructural Transformation in a Kagome Magnet

Corresponding Author: Dr Qiang Zhang

This file contains all reviewer reports in order by version, followed by all author rebuttals in order by version.

Version 0:

Reviewer comments:

Reviewer #1

(Remarks to the Author)
Tsung-Han Yang, et al in their manuscript NCOMMS-25-70324 “Intrinsic Topological Weyl Phase Transition Induced by a Magnetostructural Transformation in a Kagome Magnet”, employing high-resolution neutron diffraction and magnetization characterization experiments as well as first-principles calculations, demonstrated that Mn3Ga undergoes a chiral antiferromagnetic transition below TN1 = 485 K, followed by a magnetostructural transition (MST) to a monoclinic structure with highly canted antiferromagnetic order at a temperature TN2 = 290 K. These changes in both the crystal structure and magnetic symmetries result in an intrinsic transition between two distinct Weyl states, namely from a type-II Weyl semimetal state to another Weyl state, where the two topological states exhibit substantially different anomalous Hall conductivities.

Previous experiments [Refs. 21-24] have reported the MST transition of Mn3Ga from the high-temperature 120 chiral antiferromagnetic order below TN1 = 480 K to either the orthorhombic structure at TN2 = 120 K [Ref. 24] or the monoclinic structure at TN2 =170 K. These previous reports have also discussed the MST are accompanied by a magnetic reconfiguration that results in a substantial ferromagnetic moment [Refs. 22-25]. The current work identifies that in the temperature range TN2 < T < TN1 the structure is indeed monoclinic and the type of magnetic ordering of this phase. On the other hand, all results on the distinct anomalous Hall effect (AHE) of these structures are based solely on density functional theory calculations and not experiment.

Overall, the AHE results and the intrinsic transition between two distinct Weyl phases which can be tuned with temperature are novel and interesting. However, I am bit skeptical whether the manuscript in its current form warrants publication in high impact journal such as Nature Communications. Below I list a number of concerns and points that need to be addressed and the manuscript be revised, before any further consideration.

1. Very little is discussed on the origin of the MST. In Fig. 3E the authors state that the two distortion modes (Γ+2 and Γ+5) drive the structural transition from hexagonal to the monoclinic. How was this determined? From experiment or theoretical calculations? Is the MST transition associated only with the phonons or do the magnetic degrees of freedom play a role?

2. As a follow up of point #1 the theoretical calculations can help address the above question. Namely, they can calculate the phonon entropy contribution to the free energy as a function of temperature for both the hexagonal and monoclinic structures and investigate whether there is a structural transition between the two phases at a critical temperature. In the next step, one can add the contributions to the temperature-dependent free energy from the magnetic degrees of freedom and examine whether the critical temperature of the structural transition coincides with the corresponding of the magnetic reconfiguration.

3. In addition, since the Mn concentration is 2.94, the presence of vacancies (different annealing temperatures) and hence charge doping might affect the structural and magnetic transition temperatures and the MST. Perhaps this may be a possibility on the different value of TN2 compared to the values in Refs. 24 and 22,23. Presumably, TN2 decreases with increasing Mn deficiency?

4. The manuscript would be substantially strengthened if in addition to the magnetization measurements there were transport measurement to corroborate the different anomalous Hall effect in the two structures. The fact that there is a net magnetic moment in the monoclinic structure that would enhance the AHE.

5. Similarly, ARPES measurements can confirm the difference of location of Fermi arcs and Weyl points between the two structures.

6. The density functional theory calculations should not have assumed the structural parameters (lattice constant, angles, etc.) determined from experiment for the two structures. It would be more interesting to predict the lattice parameters from scratch for the two structures. This will also provide valuable information on the energy difference between the two structures at zero temperature.

7. How did the authors choose the nine magnetic structures shown in Fig. S4 in the supplement?

8. How do the calculated values of AHC for the hexagonal structure compare with those of previous calculations?

Reviewer #2

(Remarks to the Author)
This manuscript presents neutron diffraction, magnetization, and DFT studies on Mn3Ga, claiming the discovery of an intrinsic topological Weyl phase transition induced by a magnetostructural transformation near room temperature. While the experimental determination of the structural and magnetic transitions is technically solid and of high quality, the central topological claim is not substantiated by direct experimental evidence. My main concerns are as follows:

1. The main outcome of the study is the refinement of a magnetostructural transition from a chiral antiferromagnetic hexagonal phase to a canted antiferromagnetic monoclinic phase, accompanied by theoretical predictions of changes in Weyl-node topology and anomalous Hall conductivity. However, similar magnetostructural transitions (Refs. 22-24) and anomalous or topological Hall responses (Refs. 24, 25) in Mn3Ga have already been reported. The present work provides a more refined structural model but does not reveal fundamentally new physical phenomena.

2. The claimed Weyl phase transition is entirely based on theoretical calculations and remains speculative in nature. No transport or spectroscopic measurements (such as AHE or ARPES) are presented to validate the proposed topological reconstruction.

Therefore, while the study is technically sound and of interest to the condensed-matter community, it does not reach the level of novelty or impact expected for publication in Nature Communications. In addition, I have several further technical questions and concerns as outlined below:

3. The temperature dependence of the lattice parameters shown in Fig. 2B and Fig. 3C is not very intuitive. It is unclear what the y-axis label “ΔI/I0” represents. In Fig. 2B, the authors show that the lattice parameter aH = bH exhibits an anomaly around TN1, while in Fig. 3C the parameters aM and bM begin to deviate near TN2.

4. The evidence supporting the choice of the magnetic space group Cm’cm’ (BNS 63.464) over the previously reported P63’/m’mc’ (BNS 194.269) model appears insufficient. The authors state that refinements using Cm’cm’ provide better agreement with the neutron diffraction data, but this seems to rely mainly on minor differences in the fitting intensity (Fig. S1). In contrast, the structural distinction discussed later---where the hexagonal 300 peak splits into two reflections in the orthorhombic model and three in the monoclinic model---is more definitive. The observation of three peaks therefore favors the monoclinic model. Stronger and more direct evidence is needed to justify the assignment of Cm’cm’.

5. The significant increase of TN2 to 290 K compared with earlier reports (120–170 K) is intriguing. The authors attribute this to improved crystallinity and stoichiometry. However, it is unclear why TN1 (~480 K) remains largely unchanged within the framework of dilute magnetic moments.

Reviewer #3

(Remarks to the Author)
This manuscript shows an intrinsic magnetostructural transition (MST) between two distinct Weyl states occurs in MnGa3 at 290 K using a combination of neutron diffraction, magnetisation and first-principle calculations. The novel aspect is that the MST between these Weyl states in MnGa3 occurs without the need for external stimuli. Overall, I think the manuscript is very well presented and written, it is clear the authors have performed very good experiments and analysed their data thoroughly. I have only a few comments and questions which I think would help clarify some points.

1. As highlighted by the authors, their MnGa3 sample has a significantly higher MST than others reported previously. The authors do make some suggestions as to why including crystallinity, variations in synthesis method and stoichiometry. I find it unlikely to be stoichiometry as the sample from Ref. 23 also has 74 % Mn but only a TN of 120 K. I’m particularly interested in ball-milling, do the authors have a comparison of a sample annealed with and without ball-milling (eg. magnetisation), that could be included in the supplementary information? I think ultimately this is a really important part of this paper (partly because the sample used in this study puts the MST near room-temperature) and previous studies of MnGa3 have shown that even if the MST occurs at the same temperature, eg. 170 K, then the low-temperature nuclear structure can be different.

2. Additionally, ball-milling the sample has been known to decrease the particle size, which in turn can cause strain and significantly increase the peak width in diffraction – did the authors see any evidence for peak broadening and if so how did

they account for this in their refinements?

3. In Figure 2, there is a star above the main peak in panels d and e. I’m assuming that’s the 101 peak, but if it could be clarified in the caption that would be helpful. There is also a star used in panel a for MnO, which I think adds to the confusion, so perhaps the authors could use a different symbol for the impurity.

4. On page 9, table 1 and page 13, table 2, it is not clear to me how the authors have calculated |M|. Please could the authors add an explanation to the methods section.

5. On page 11, from the Rietveld refinement at 30 K, it first says there is a substantial net moment of 2.14 μB, and then later on in the paragraph, it says the ordered moment per Mn site is 2.56 μB. What is the reason for this discrepancy?

Version 1:

Reviewer comments:

Reviewer #1

(Remarks to the Author)
The authors responded satisfactorily to all my comments and criticisms and have revised the manuscript accordingly. They have performed additional comprehensive transport measurements of the anomalous Hall effect (AHE) and the longitudinal resistivity which revealed very distinct AHE between the two crystalline and magnetic phases with opposite signs and different magnitudes below TN2. These additional results have strengthened the manuscript immensely.

Consequently, the manuscript warrants publication in Nature Communications in its present form.

Reviewer #2

(Remarks to the Author)
The authors have made extensive efforts to address the previous concerns by performing major revisions and adding comprehensive transport measurements. I am satisfied with these revisions. The manuscript is now significantly strengthened and presents a more complete and convincing study combining neutron diffraction, transport experiments, and DFT calculations. The magnetostructural transition from a chiral antiferromagnetic hexagonal phase to a canted antiferromagnetic monoclinic phase is well established. Moreover, the electronic band structure calculations reveal distinct Weyl states in the two phases, which are accompanied by pronounced changes in the anomalous Hall effect and the emergence of a topological Hall effect in the transport measurements. These new experimental results provide strong support for the claimed topological Weyl phase transition. Therefore, I recommend that this work be published in Nature Communications.

Reviewer #3

(Remarks to the Author)
I would like to thank the authors for making the changes I suggested. I support the manuscript for publication in Nature Communications.

Open Access This Peer Review File is licensed under a Creative Commons Attribution 4.0 International License, which permits use, sharing, adaptation, distribution and reproduction in any medium or format, as long as you give appropriate credit to the original author(s) and the source, provide a link to the Creative Commons license, and indicate if changes were made.
In cases where reviewers are anonymous, credit should be given to 'Anonymous Referee' and the source.
The images or other third party material in this Peer Review File are included in the article's Creative Commons license, unless indicated otherwise in a credit line to the material. If material is not included in the article's Creative Commons license and your intended use is not permitted by statutory regulation or exceeds the permitted use, you will need to obtain permission directly from the copyright holder.
To view a copy of this license, visit https://creativecommons.org/licenses/by/4.0/

Reply to the referees' comments on manuscript # NCOMMS-25-70324

We express our sincere appreciation for all the referees' very constructive comments. We have taken these comments into account fully and have revised our manuscript accordingly.

Here are our responses and the corresponding revisions for each of the comments:

Reviewer #1 (Remarks to the Author):

Comment:

Tsung-Han Yang, et al in their manuscript NCOMMS-25-70324 "Intrinsic Topological Weyl Phase Transition Induced by a Magnetostructural Transformation in a Kagome Magnet", employing high-resolution neutron diffraction and magnetization characterization experiments as well as first-principles calculations, demonstrated that Mn_3Ga undergoes a chiral antiferromagnetic transition below T_{N1} = 485 K, followed by a magnetostructural transition (MST) to a monoclinic structure with highly canted antiferromagnetic order at a temperature T_{N2} = 290 K. These changes in both the crystal structure and magnetic symmetries result in an intrinsic transition between two distinct Weyl states, namely from a type-II Weyl semimetal state to another Weyl state, where the two topological states exhibit substantially different anomalous Hall conductivities.

Previous experiments [Refs. 21-24] have reported the MST transition of Mn_3Ga from the high-temperature 120 chiral antiferromagnetic order below T_{N1} = 480 K to either the orthorhombic structure at T_{N2} = 120 K [Ref. 24] or the monoclinic structure at T_{N2} =170 K. These previous reports have also discussed the MST are accompanied by a magnetic reconfiguration that results in a substantial ferromagnetic moment [Refs. 22-25]. The current work identifies that in the temperature range $T_{N2} < T < T_{N1}$ the structure is indeed monoclinic and the type of magnetic ordering of this phase. On the other hand, all results on the distinct anomalous Hall effect (AHE) of these structures are based solely on density functional theory calculations and not experiment.

Overall, the AHE results and the intrinsic transition between two distinct Weyl phases which can be tuned with temperature are novel and interesting. However, I am bit skeptical whether the manuscript in its current form warrants publication in high impact journal such as Nature Communications. Below I list a number of concerns and points that need to be addressed and the manuscript be revised, before any further consideration.

Reply:

We thank the reviewer for recognizing the novel and interesting results in the manuscript and for providing very constructive comments to help us improve our work. Following the reviewer's comments, we have added new transport and magnetization hysteresis loop experiments, added two new sections, and made major revisions to the manuscript, as detailed below. These revisions not only provide experimental evidence of topological reconstruction but also identify an emergent anomalous phase driven by spin chirality at low temperatures and fields.

In addition, we would like to explain that determining the correct crystal and magnetic structures below T_{N2} in Mn_3Ga was a challenge and the low-T magnetic structure below in $T<T_{N2}$ presented in our manuscript differs significantly from that proposed in previous reports. We found that neutron powder diffraction alone cannot unambiguously determine the magnetic structure in Mn_3Ga due to overlapping nuclear and magnetic peaks and the subtlety of the simultaneous lowering of crystalline and magnetic symmetry. Two magnetic models A and H shown in Fig. S7 of SI yield similar refinement quality on the neutron data. To resolve this, we combined high-resolution neutron diffraction with DFT calculations on the energies to determine the correct magnetic structure. Additionally, the crystal structure of Mn_3Ga has been controversial, with debates between orthorhombic and monoclinic configurations. Although Ref. 23 claimed a monoclinic structure, only a splitting into two peaks (consistent with orthorhombic symmetry) was observed, and there was no evidence for the three-peak splitting expected from a monoclinic distortion. This likely explains why subsequent studies, such as Ref. 25 in *Scientific Reports*, continued to adopt an orthorhombic model. To

resolve this, we employed a high-resolution setup of neutron diffraction that revealed a three-peak splitting from the hexagonal 300 peak, with excellent refinement quality, providing strong evidence for a transition to a monoclinic structure.

Thus, determining the correct crystal and magnetic structures below T_{N2} in Mn_3Ga was difficult. We think the crystal and magnetic structures presented in our manuscript are new and important, forming a crucial foundation for exploring the topological Weyl states and the anomalous Hall effect in Mn_3Ga. In particular, the specific magnetic symmetry below T_{N2} is required to protect the Type-I Weyl nodes shown in our DFT calculations, directly linking the structural solution to the new topological physics.

We apologize that this was not clear in previous manuscript and we have clarified this in page 12 of the revised manuscript.

1. Very little is discussed on the origin of the MST. In Fig. 3E the authors state that the two distortion modes (Γ+2 and Γ+5) drive the structural transition from hexagonal to the monoclinic. How was this determined? From experiment or theoretical calculations? Is the MST transition associated only with the phonons or do the magnetic degrees of freedom play a role?

Reply:

Thanks for the insightful comments. The specific distortion modes (Γ2+ and Γ5+) were determined through a rigorous group-theoretical analysis of our experimental diffraction data, using the STRUCTURE RELATIONS tool available on the Bilbao Crystallography Server (BCS).

We provided the experimentally determined crystal structures of the high-temperature hexagonal (parent) phase and the low-temperature monoclinic (child) phase as input. The STRUCTURE RELATIONS tool on the BCS then decomposes the total structural distortion into its symmetry-allowed irreducible representations (irreps) and calculates their respective amplitudes. This analysis unambiguously identified Γ2+ and Γ5+ as the active modes responsible for the transition.

This directly addresses the second part of the question. The fact that this structural transition coincides precisely with the magnetic transition at T_{N2} is strong evidence that this is a magnetostructural transition (MST). The magnetic degrees of freedom are not just involved; they are the likely primary driving force. The structural distortion (via the Γ2+ and Γ5+ modes) acts as a secondary order parameter that couples to the primary magnetic order. This is a classic case of strong spin-lattice (or magnetoelastic) coupling, where the lattice distorts to accommodate the new magnetic ground state, likely to relieve magnetic frustration present in the high-symmetry hexagonal phase.

We have made the revisions in page 10 in the main text to clarify that the distortion modes were obtained from symmetry analysis based on experimentally refined model.

2. As a follow up of point #1 the theoretical calculations can help address the above question. Namely, they can calculate the phonon entropy contribution to the free energy as a function of temperature for both the hexagonal and monoclinic structures and investigate whether there is a structural transition between the two phases at a critical temperature. In the next step, one can add the contributions to the temperature-dependent free energy from the magnetic degrees of freedom and examine whether the critical temperature of the structural transition coincides with the corresponding of the magnetic reconfiguration.

Reply:

We thank the reviewer very much for this comment, which is related to comment 6. Since we use experimental lattice parameters for both the low-temperature monoclinic phase and the high-temperature hexagonal phase, the computed force constants are not reliable to evaluate phonon dispersions and the phonon contribution to the temperature-

dependent free energy. Instead, in the revised manuscript, we have included the calculated total energy difference between the two relevant phases in page 22: the low-temperature monoclinic structure with its stable magnetic ordering is approximately 0.041 eV ~ 475 K lower in energy than the high-temperature hexagonal structure (also with its stable ordering). Thermal effects, such as the reduction in ordered magnetic moments and lattice fluctuation, may reasonably account for the experimentally observed magnetostructural transition between the two phases.

3. In addition, since the Mn concentration is 2.94, the presence of vacancies (different annealing temperatures) and hence charge doping might affect the structural and magnetic transition temperatures and the MST. Perhaps this may be a possibility on the different value of T_{N2} compared to the values in Refs. 24 and 22,23. Presumably, T_{N2} decreases with increasing Mn deficiency?

Reply:

We thank the reviewer for this comparison. Our samples have a higher Mn concentration (~74.6 at%) compared to the 70–74 at% reported in Refs. 22–24. This higher Mn content correlates with a substantial increase in T_{N2} to 295 K, up from the previously reported ~150 K. This confirms the trend that T_{N2} decreases with increasing Mn deficiency as the reviewer pointed out, likely due to the dilution of magnetic exchange interactions.

4. The manuscript would be substantially strengthened if in addition to the magnetization measurements there were transport measurement to corroborate the different anomalous Hall effect in the two structures. The fact that there is a net magnetic moment in the monoclinic structure that would enhance the AHE.

Reply:

We fully agree with reviewer that the experimental evidence of the anomalous Hall effects in both structures is important. Following reviewer's comment, we have performed comprehensive transport measurements including the anomalous Hall effect ρ_{yx} and longitudinal resistivity ρ_{xx}. New results have been added in Figs 7, 8 and Supplementary Fig. 5, along with a detailed discussion in the newly added section titled "Transport Signatures of the Topological Phase Transition" in page 16–21. We observed a very distinct Anomalous Hall Effect (AHE) between the two crystalline and magnetic phases, with opposite signs and different magnitudes below T_{N2}, accompanied by the emergence of topological Hall effect (THE) below ~ 150 K.

As pointed out by the referee, the net magnetic moment in the monoclinic structure indeed enhanced the AHE, leading to a two-fold increase in the maximum anomalous Hall conductivity (AHC). Since the change in the AHC is closely related to the change of integrated Berry curvature with the redistribution of Weyl nodes, these transport signatures provide robust experimental evidence supporting our theoretically predicted topological reconstruction in the absence of ARPES-grade crystals.

Furthermore, an anomalous phase characterized by spin chirality is identified at low fields below approximately 150 K as indicated by the appearance of THE. A new discussion section titled "Emergent Anomalous Phase at Low Temperatures and Fields" has been added in page 22-23. Additionally, revisions have been made to the "Discussion" section on pages 21–23.

5. Similarly, ARPES measurements can confirm the difference of location of Fermi arcs and Weyl points between the two structures.

Reply:

We fully agree that ARPES would provide direct visualization of the Fermi arcs. However, it requires single crystal form of Mn_3Ga for such measurements. We have only succeeded in synthesizing polycrystalline samples exhibiting high T_{N2} near room temperature. Getting nearly stoichiometric Mn_3Ga single crystal with comparably high T_{N2} is very

challenging. Nevertheless, we believe our results on polycrystalline samples will motivate single crystal growth and subsequent ARPES measurements.

6. The density functional theory calculations should not have assumed the structural parameters (lattice constant, angles, etc.) determined from experiment for the two structures. It would be more interesting to predict the lattice parameters from scratch for the two structures. This will also provide valuable information on the energy difference between the two structures at zero temperature.

Reply:

We appreciate the reviewer's suggestion regarding a comprehensive search over all lattice and magnetic degrees of freedom.

We fully agree that the suggested procedure is ideal for a global search. Nevertheless, , its application to this material is computationally very challenging. First, a high degree of freedom could induce multiple local energy minima. Identifying the true global ground state (the most stable structure) in the resulting complex energy landscape would be exceedingly difficult and computationally expensive. Second, the relative stability of different structural and magnetic orderings can be highly sensitive to specific details of the DFT calculation, such as the chosen exchange-correlation functional or the application of the +U correction. Considering these constraints, we adopted a pragmatic and experimentally validated approach. We utilized the experimentally determined low-temperature monoclinic crystal structure as our starting point. We then systematically examined the stability of nine distinct initial magnetic configurations within this fixed structure. Although this approach does not guarantee finding the absolute global minimum, the most stable magnetic ordering we identified is consistent with our experimental observations. This agreement provides significant confidence in the reliability and physical relevance of our DFT results.

We have made the revisions in page 22 in the main text and SI to provide more details.

7. How did the authors choose the nine magnetic structures shown in Fig. S4 in the supplement?

Reply:

We thank the reviewer for this comment. We revised the corresponding paragraph in the supplementary information by providing the schematics of initial magnetic configurations. A new Supplementary Fig. 6 was added.

8. How do the calculated values of AHC for the hexagonal structure compare with those of previous calculations?

Reply:

To the best of our knowledge, there are no previous reports on the calculated anomalous Hall conductivity (AHC) for the hexagonal structure of Mn_3Ga.

Reviewer #2 (Remarks to the Author):

This manuscript presents neutron diffraction, magnetization, and DFT studies on Mn_3Ga, claiming the discovery of an intrinsic topological Weyl phase transition induced by a magnetostructural transformation near room temperature. While the experimental determination of the structural and magnetic transitions is technically solid and of high quality, the central topological claim is not substantiated by direct experimental evidence. My main concerns are as follows:

Reply:

We thank the referee for summarizing the main message of our work and agreeing on the high quality of our experimental determination of the structural and magnetic transitions. We fully agree with the referee that lacking direct experimental evidence supporting the topological claim was the weakness of our original manuscript. Following the referee's comments, we have added transport measurements for both anomalous hall effect ρ_{yx} and longitudinal resistivity ρ_{xx}, as well as corresponding magnetization hysteresis loops at the same temperatures. These revisions not only provide experimental evidence of topological reconstruction but also identify an emergent anomalous phase driven by spin chirality at low temperatures and fields.

1. The main outcome of the study is the refinement of a magnetostructural transition from a chiral antiferromagnetic hexagonal phase to a canted antiferromagnetic monoclinic phase, accompanied by theoretical predictions of changes in Weyl-node topology and anomalous Hall conductivity. However, similar magnetostructural transitions (Refs. 22-24) and anomalous or topological Hall responses (Refs. 24, 25) in Mn_3Ga have already been reported. The present work provides a more refined structural model but does not reveal fundamentally new physical phenomena.

Reply:

We thank the reviewer for the comment on the novelty of our work. After considering the referee's comments, we have added new transport experiments, data analysis and discussion in the revised manuscript. We believe that the revised manuscript has been improved significantly. Below, we explain the advances and novel aspects of our revised manuscript compared to previous reports.

(1) We discovered Mn_3Ga as the first example exhibiting an intrinsic topological Weyl phase transition between two distinct Weyl states, driven by a magnetostructural transition. This has never been reported in Mn_3Ga or other materials.

By combining our magnetic hysteresis loops, precise magnetic structures, experimental AHE data, and DFT calculations on both AHE and Weyl states, we have not only validated the topological reconstruction but also identified an anomalous phase driven by spin chirality at low temperatures and fields. The emerging topological Hall effect, coexisting with the AHE at low temperatures, highlights a remarkable unification between the real-space Berry phase from spin chirality and the k-space Berry curvature inherent in the band topology.

Although anomalous Hall Effect have been reported in their samples in Refs. 24, 25, they were not correlated to topological Weyl states. Furthermore, there are key differences between our work and previous results:

a) Below T_{N2}, while Refs. 24 and 25 report the AHE in an orthorhombic structure, our results indicate that both the AHE and THE are associated with a distinct monoclinic structure.
b) A sign change in the longitudinal magnetoresistance (MR) was observed only in our sample.
c) We extracted distinct contributions from the anomalous Hall effect (AHE) with opposite signs and different magnitudes to support the topological reconstruction, with the effect occurring near room temperature—significantly higher than the approximately 140 K reported in Ref. 25. No sign change of the AHE was observed in Ref. 24.
d) The topological Hall effect (THE) was found at a higher temperature of ~150 K in our study compared to ~100 K in Ref. 25.

(2) Determining the correct crystal and magnetic structures below T_{N2} in Mn_3Ga was a significant challenge, which we resolved by combining neutron diffraction and DFT calculations. The crystal structure of Mn_3Ga has been controversial, with debates between orthorhombic and monoclinic configurations. Although Ref. 23 claimed a monoclinic structure, only a splitting into two peaks (consistent with orthorhombic symmetry) was observed, and there was no evidence for the three-peak splitting expected from a monoclinic distortion. This likely explains why subsequent studies, such as Ref. 25 in *Scientific Reports*, continued to adopt an orthorhombic model. To resolve this, we employed a high-resolution setup of neutron diffraction that revealed a three-peak splitting from the hexagonal 300 peak, with excellent refinement quality, providing strong evidence for a transition to a monoclinic structure.

Furthermore, we solved a new magnetic structure and determined the net moment direction. This magnetic structure differs significantly from that proposed in Ref. 23. Note that neutron powder diffraction alone cannot unambiguously determine the magnetic structure in Mn_3Ga overlapping nuclear and magnetic peaks and the subtlety of the simultaneous lowering of crystalline and magnetic symmetry. Two magnetic models, A and H shown in Fig.S7 of SI yield similar excellent refinement quality. To resolve this, we combined high-resolution neutron diffraction with DFT calculations to determine the correct magnetic structure. Thus, it turns out that determining the correct crystal and magnetic structures below T_{N2} in Mn_3Ga was a challenging task. In particular, the low-T magnetic structure below in $T<T_{N2}$ presented in our manuscript differs significantly from that proposed in previous reports. This specific magnetic symmetry is required to protect the Type-I Weyl nodes shown in our DFT calculations, directly linking the structural solution to the new topological physics. We therefore think the crystal and magnetic structures presented in our manuscript are new and important, providing a crucial foundation for exploring the topological Weyl states and the anomalous Hall effect in Mn_3Ga.

We apologize that this was not clear in previous manuscript and we have revised this part in page 12 of the revised manuscript.

(3) Room-Temperature Functionality: Our stoichiometric control stabilizes the Weyl phase transition near room temperature, facilitating the exploration of potential spintronic applications through the manipulation of two distinct Weyl states.

2. The claimed Weyl phase transition is entirely based on theoretical calculations and remains speculative in nature. No transport or spectroscopic measurements (such as AHE or ARPES) are presented to validate the proposed topological reconstruction.

Reply:

We thank the reviewer for these important comments. We agree that experimental validation of topological reconstruction is essential. To address this, we have performed comprehensive temperature-dependent transport measurements for both anomalous Hall effect ρ_{yx} and longitudinal resistivity ρ_{xx}, as well as corresponding magnetization hysteresis loops at the same temperatures. By combining experimental AHE and DFT calculations on AHE and Weyl states, we are able to validate the proposed topological reconstruction.

We observed a very distinct Anomalous Hall Effect (AHE)–with opposite signs and different magnitudes—between the two crystalline and magnetic phases, together with the emergence of topological Hall effect below T_{N2}. These transport signatures provide strong experimental evidence for the proposed topological transition. Furthermore, our DFT calculations [Fig. 5] show that the high-symmetry hexagonal phase restricts the Hall tensor (only $\sigma_{zx}\neq0$), whereas the low-symmetry monoclinic phase allows additional components (e.g., σ_{yz}) related to the reorganization of Weyl nodes from Type-II to Type-I, resulting in an enhanced anomalous Hall conductivity (AHC). The observed two-fold increase of the AHC at low temperatures below T_{N2} is consistent with this theoretical prediction. In addition, an anomalous phase due to spin chirality is identified at low fields below approximately 150 K, as indicated by the appearance of THE.

We agree that ARPES would provide direct visualization of the Fermi arcs. However, it requires single crystal form of Mn_3Ga for such measurements. We have only succeeded in synthesizing polycrystalline samples exhibiting high T_{N2} near room temperature. Getting nearly stoichiometric Mn_3Ga single crystal with comparably high T_{N2} is very challenging. We are sorry that we are unable to present the ARPES measurements. Nevertheless, we believe that the dramatic changes in the experimental AHE and THE are sufficient to evidence our theoretically predicted topological reconstruction in the absence of ARPES-grade crystals.

We have added two sections of "Transport Signatures of the Topological Phase Transition" and ""Emergent anomalous phase at low temperatures and fields" in pages 17-21, two new Figs. 7 and 8 in page 17 and 19, as well as Supplementary Fig. 5. Additionally, major revisions have been made to the "Discussion" section on pages 21–23.

Comments: Therefore, while the study is technically sound and of interest to the condensed-matter community, it does not reach the level of novelty or impact expected for publication in Nature Communications. In addition, I have several further technical questions and concerns as outlined below:

Reply:

To address the referee's concern regarding the novelty of the original manuscript, we have made extensive efforts by adding new experiments and performing major revisions to significantly enhance the quality of our work, as detailed above. We have also provided point-by-point responses to the other technical questions and concerns raised by the referee below. Hopefully, the referee could be convinced that our revised manuscript is now suitable for publication in *Nature Communications*.

3. The temperature dependence of the lattice parameters shown in Fig. 2B and Fig. 3C is not very intuitive. It is unclear what the y-axis label "$\Delta l/l_0$" represents. In Fig. 2B, the authors show that the lattice parameter $a_H = b_H$ exhibits an anomaly around T_{N1}, while in Fig. 3C the parameters a_M and b_M begin to deviate near T_{N2}.

Reply:

We thank the reviewer for pointing out this ambiguity.

(1) Clarification of y-axis label: The y-axis represents the relative change in lattice parameters, defined as [l(T)–l(300K)]/l(300K). We have added the description of the definition in the caption of Fig. 3 (C–D).
(2) Relationship between Fig. 2(B) and Fig. 3(C): In Fig. 2(B), the hexagonal cell parameters ($a_H = b_H$) show an anomaly at T_{N1}. But the structure remains hexagonal across T_{N1}. In contrast, below T_{N2}, the lattice constant a_H splits into distinct a_M and c_M values, indicating a structural transition from hexagonal to monoclinic symmetry.

We have clarified this in the figure caption and text to make the relationship between the two representations explicit.

4. The evidence supporting the choice of the magnetic space group Cm'cm' (BNS 63.464) over the previously reported P63'/m'mc' (BNS 194.269) model appears insufficient. The authors state that refinements using Cm'cm' provide better agreement with the neutron diffraction data, but this seems to rely mainly on minor differences in the fitting intensity (Fig. S1). In contrast, the structural distinction discussed later---where the hexagonal 300 peak splits into two reflections in the orthorhombic model and three in the monoclinic model---is more definitive. The observation of three peaks therefore favors the monoclinic model. Stronger and more direct evidence is needed to justify the assignment of Cm'cm'.

Reply:

We appreciate the reviewer's comment and the comparison to the structural analysis. We combined both Rietveld analysis and DFT calculations to distinguish *Cm'cm'* and *P63'/m'mc'*.

(1) To determine ($k = 0$) magnetic order, the high-resolution neutron diffraction data with wide Q coverage is essential to obtain the reliable structural parameters, such as the scale factor, atomic positions, thermal parameters, occupancy, etc., for separating the nuclear and magnetic contributions to the same peak and distinguishing different magnetic models. Our neutron data covered a wide d spacing so that we can get reliable structural parameters by the fits to a lot of middle-d and high-d parts of the neutron data with negligible contribution from magnetic peaks as shown in the new Fig. S2(B) figure (only high-Q data shown for clarity). Then, we fixed these structural parameters and only refined magnetic moments for these two magnetic models and compare the relative intensities of the characteristic magnetic peaks. As shown in Fig. S2(a,c), the refinement quality for the characteristic ratio I(100)/I(110) is well reproduced using the *Cm'cm'*, but not by the *P63'/m'mc'*.

(2) Our DFT calculations reveal that the energy for the *Cm'cm'* configuration is −30.002 eV /f.u., which is lower than the -29.996 eV /f.u obtained for the *P 63'/m'mc'* configuration.

The consistency between our neutron diffraction and DFT calculations validated the *Cm'cm'* spin configuration.
We have made the revisions in page 7–9 in the main text, and Supplementary Fig. S2 in the Supporting Information.

5. The significant increase of T_{N2} to 290 K compared with earlier reports (120–170 K) is intriguing. The authors attribute this to improved crystallinity and stoichiometry. However, it is unclear why T_{N1} (~480 K) remains largely unchanged within the framework of dilute magnetic moments.

Reply:

We really appreciate the reviewer for this insightful comment, which motivated us to do a more thorough review of previous reports on T_{N1}. In earlier studies (Ref. 21 and 22), T_{N1} was reported to be approximately 460 K and 470 K, respectively. To be more accurate, the T_{N1} in previous reports was below ~470 K, while our sample exhibits a slight increase of approximately 15 K. The Néel temperature in Mn_3X (X=Ga, Sn or Ge) is governed by strong Mn–Mn exchange interactions. Variations in stoichiometry typically lead to modifications in the lattice constants and Mn–Mn bond lengths, which is expected to modify Mn–Mn exchange interactions and therefore, induces a slight change of T_{N1}.

In contrast, T_{N2} corresponds to a magnetostructural transition where both the crystal structure and magnetic order change simultaneously and are intimately coupled. Consequently, even small variations in lattice parameters/magnetic interactions can induce a much larger shift in T_{N2}. This high sensitivity to perturbations such as composition and preparation conditions has been observed in not only Mn_3Ga but other samples undergoing magnetostructural transitions, as discussed in the section "Engineering Magnetostructural Transitions" in page 24-25.

We have updated the previously reported T_{N1} from 480 K to a more accurate 470 K in the "Introduction" part and added one sentence on page 6-7 of the main text to comment on the difference in T_{N1}.

Reviewer #3 (Remarks to the Author):

This manuscript shows an intrinsic magnetostructural transition (MST) between two distinct Weyl states occurs in Mn_3Ga at 290 K using a combination of neutron diffraction, magnetisation and first-principle calculations. The novel aspect is that the MST between these Weyl states in MnGa3 occurs without the need for external stimuli. Overall, I think the manuscript is very well presented and written, it is clear the authors have performed very good experiments and analysed their data thoroughly. I have only a few comments and questions which I think would help clarify some points.

Reply:

We thank the reviewer for recognizing the novelty of our manuscript and for the valuable comments very much. We have responded to these comments one-by-one and made corresponding revisions to the manuscript.

1. As highlighted by the authors, their Mn_3Ga sample has a significantly higher MST than others reported previously. The authors do make some suggestions as to why including crystallinity, variations in synthesis method and stoichiometry. I find it unlikely to be stoichiometry as the sample from Ref. 23 also has 74 % Mn but only a T_N of 120 K. I'm particularly interested in ball-milling, do the authors have a comparison of a sample annealed with and without ball-milling (eg. magnetisation), that could be included in the supplementary information? I think ultimately this is a really important part of this paper (partly because the sample used in this study puts the MST near room-temperature) and previous studies of Mn_3Ga have shown that even if the MST occurs at the same temperature, eg. 170 K, then the low-temperature nuclear structure can be different.

Reply:

We thank the reviewer for this incisive observation. As suggested, we have performed a direct comparative study, now presented in the new Figure S1 of the Supplementary Information, which contrasts the magnetization of samples synthesized with and without ball-milling. It seems that the sample with additional ball-milling process shows a lower transition temperature by approximately 20 K, whereas the sample without ball milling exhibits a higher T_{N2} ~ 320 K.

The reviewer's comment motivated us to do more thorough literature reviews and experiments. For example, In Ref. 22, a sample with 71.4 at% Mn shows a T_{N2} of 170 K. In Ref. 23, the composition of the sample is $Mn_{2.9}Ga$ (~74 % Mn) showing a low T_{N2} of 120 K as the referee pointed out. We noticed that Ref. 23 also mentioned another composition, $Mn_{2.95}Ga$ (~74.7 at % Mn) exhibiting a high T_{N2} of ~285 K, which is close to our study ($Mn_{2.94}Ga$,~74.6 at % Mn; T_{N2}: 295-320 K). All these results indicate that T_{N2} is highly sensitive to the stoichiometry in the region ~71 -75 am% Mn.

However, we agree that the stoichiometry alone is not sufficient to account for the increase of T_{N2} in our sample. Factors such as synthesis methods, annealing temperature, crystallinity, and other processing conditions also play some role. Our new test showed that additional round of annealing period of 6 hours after the quenching process reduced T_{N2} by around 10 K.

We have added more information in page 10, 26 of the main text and added new S1 figure in the Supporting Information (SI).

2. Additionally, ball-milling the sample has been known to decrease the particle size, which in turn can cause strain and significantly increase the peak width in diffraction – did the authors see any evidence for peak broadening and if so how did they account for this in their refinements?

Reply:

We thank the reviewer for raising this important point regarding sample microstructure. We agree that ball-milling typically induces mechanical strain and reduces particle size, which can lead to significant peak broadening.

To avoid this, we did not use a high-energy ball milling process. Instead, we employed a low-energy ball milling technique solely to pulverize the ingots into powder. A critical post-milling annealing step was then performed at 873 K for 17 hours followed by a quenching process. This procedure effectively relieved the mechanical strain and promoted grain growth/recrystallization. Consequently, the neutron diffraction patterns exhibit very sharp Bragg peaks, as shown in Fig.2(d-e). The peak widths are dominated by instrumental resolution, and we did not observe clear broadening beyond instrument resolution. In our GSAS-II refinements, instrumental resolution parameters were sufficient to model the peak shapes, and no additional microstrain term was required.

3. In Figure 2, there is a star above the main peak in panels d and e. I'm assuming that's the 101 peak, but if it could be clarified in the caption that would be helpful. There is also a star used in panel a for MnO, which I think adds to the confusion, so perhaps the authors could use a different symbol for the impurity.

Reply:

We apologize for the confusion in Fig. 2. We have revised it and now use a different symbol to clearly distinguish the MnO impurity phase.

4. On page 9, table 1 and page 13, table 2, it is not clear to me how the authors have calculated |M|. Please could the authors add an explanation to the methods section.

and

5. On page 11, from the Rietveld refinement at 30 K, it first says there is a substantial net moment of 2.14 μB, and then later on in the paragraph, it says the ordered moment per Mn site is 2.56 μB. What is the reason for this discrepancy?

Reply:

|M| is the absolute ordered moment and defined from the vector **M**=(m_x, m_y, m_z). Thus, |M| in Mn_3Ga was calculated based on the two moment components of (mx, my, 0) in the hexagonal phase or (mx, 0, mz) in the monoclinic phase, incorporating the respective inter-component angles. And the GSAS-II software computed |M| automatically, which can be found in the output .lst file.

The net moment and ordered moment per Mn site refer to different quantities. The ordered moment of 2.56 μB/Mn site was obtained from the magnetic Rietveld refinement. In sharp contrast, the value of 2.14 μB is the total net moment in one triangular Mn lattice, i.e., the vector sum of all the three Mn-site moments. To avoid confusion, we change to use average net moment on a per-Mn basis as 2.14/3≈0.71 μB/Mn. Because the refined structure is non-collinear, the individual Mn moments partially cancel, so this value is smaller than the ordered moment per Mn.

In addition, we have added a couple sentences on page 27 explaining how |M| and net moment are obtained and clarified the difference between net moment and ordered moment in the methods section.

List of main changes:

1. Revised Abstract, Introduction and Conclusion parts to add new findings.
2. Page 6, Figure 2(A): Changed the symbol of MnO for clarity.
3. Page 8, Figure 3(C–D): Added the description of the definition of normalized lattice constants and the cell angle for clarity.
4. Page 7-9: Added the DFT results and discussion on the two magnetic magnetic structures in $T_{N2}<T<T_{N1}$.
5. Page 10: Added the detail of symmetry analysis for clarity.
6. Page 10: Added the influence of ball-milling on the transition temperature.
7. Page 12: Clarified the net moment for the monoclinic phase, and SI figure labels.
8. Page 12: Revised the part to explain how the low-T magnetic structure in $T<T_{N1}$ was determined by a combined use of neutron diffraction and DFT calculations.
9. Page 16-21: Added a new section titled "Transport Signatures of the Topological Phase Transition", along with new Figs 7, 8 and Supplementary Fig. 5.
10. Page 21-22: Added discussion on the driving force of the magnetostructural transition based on DFT results.
11. Page 22-23: Added a new discussion subsection titled "Emergent Anomalous Phase at Low Temperatures and Fields".
12. Page 23-24: Added discussion and new insight in section "MST-Driven Topological Weyl phase transition".
13. Page 26: Sample preparation: Added more details for sample preparation for the transport measurements.
14. Page 27: Added how |M| and net moment are obtained and clarified the difference between net moment and ordered moment.
15. Page 27-28: Add the description of the details of the new transport measurements.
16. In SI, Added new Fig. 1, 5, 6.
17. In SI: Improved Fig. 2.
18. In SI: Added how the nine magnetic configurations were chosen.
